# Exploration of Free Energy Surface and Thermal Effects on Relative Population and Infrared Spectrum of the Be_6_B_11_^−^ Fluxional Cluster

**DOI:** 10.3390/ma14010112

**Published:** 2020-12-29

**Authors:** Carlos Emiliano Buelna-Garcia, José Luis Cabellos, Jesus Manuel Quiroz-Castillo, Gerardo Martinez-Guajardo, Cesar Castillo-Quevedo, Aned de-Leon-Flores, Gilberto Anzueto-Sanchez, Martha Fabiola Martin-del-Campo-Solis

**Affiliations:** 1 Departamento de Investigación en Polímeros y Materiales, Edificio 3G, Universidad de Sonora, Blvd. Luis Encinas y Rosales S/N, Centro, Hermosillo 83000, Mexico; a209205768@unison.mx (C.E.B.-G.); jesus.quiroz@unison.mx (J.M.Q.-C.); 2 Departamento de Investigación en Fisica, Edifcio 3M, Universidad de Sonora, Blvd. Luis Encinas y Rosales S/N, Centro, Hermosillo 83000, Mexico; 3 Unidad Académica de Ciencias Químicas, Área de Ciencias de la Salud, Universidad Autónoma de Zacatecas, Km. 6 Carretera Zacatecas-Guadalajara s/n, Ejido La Escondida, Zacatecas 98160, Mexico; germtzguajardo@uaz.edu.mx; 4 Departamento de Fundamentos del Conocimiento, Centro Universitario del Norte, Universidad de Guadalajara, Carretera Federal No. 23, Km. 191, Colotlán 46200, Mexico; castillo.quevedo@cunorte.udg.mx (C.C.-Q.); mfmartindelcampo@cunorte.udg.mx (M.F.M.-d.-C.-S.); 5 Departamento de Ciencias Quimico Biologicas, Edifico 5A, Universidad de Sonora, Blvd. Luis Encinas y Rosales S/N, Centro, Hermosillo 83000, Mexico; aned.deleon@unison.mx; 6 Centro de Investigaciones en Óptica, A.C., León 37150, Mexico; gilberto.anzueto@cio.mx

**Keywords:** global minimum, infrared spectrum, boron cluster, fluxional, density functional theory, temperature, Boltzmann factors, Gibbs free energy, entropy, Grimme’s approach (DFT-D3), IR spectra

## Abstract

The starting point to understanding cluster properties is the putative global minimum and all the nearby local energy minima; however, locating them is computationally expensive and difficult. The relative populations and spectroscopic properties that are a function of temperature can be approximately computed by employing statistical thermodynamics. Here, we investigate entropy-driven isomers distribution on Be_6_B_11_^−^ clusters and the effect of temperature on their infrared spectroscopy and relative populations. We identify the vibration modes possessed by the cluster that significantly contribute to the zero-point energy. A couple of steps are considered for computing the temperature-dependent relative population: First, using a genetic algorithm coupled to density functional theory, we performed an extensive and systematic exploration of the potential/free energy surface of Be_6_B_11_^−^ clusters to locate the putative global minimum and elucidate the low-energy structures. Second, the relative populations’ temperature effects are determined by considering the thermodynamic properties and Boltzmann factors. The temperature-dependent relative populations show that the entropies and temperature are essential for determining the global minimum. We compute the temperature-dependent total infrared spectra employing the Boltzmann factor weighted sums of each isomer’s infrared spectrum and find that at finite temperature, the total infrared spectrum is composed of an admixture of infrared spectra that corresponds to the spectra of the lowest-energy structure and its isomers located at higher energies. The methodology and results describe the thermal effects in the relative population and the infrared spectra.

## 1. Introduction

In recent years, pure, metal-, and non-metal-doped boron clusters have attracted considerable attention [1,2,3,4,5,6,7,8,9,10] due to their unpredictable chemistry [11,12] and high potential to form novel structures [13]. Boron is the smallest and lightest semi-metal [7,14] and a neighbor of carbon in the periodic table. It is electron-deficient [13,15,16,17] and can combine and form novel atomic and molecular boron structures that are planar and quasi-planar [18,19,20]. It can also form nanotubes [13,21], borospherenes [2,22,23], borophene [2], cages [24], chiral helices [25,26], and nanosheets [18,27,28] consisting of triangle units of boron atoms. Boron can absorb neutrons, which makes it useful for nuclear and medical applications [29,30,31,32]. In boron-based clusters, aromaticity, antiaromaticity, and conflicting aromaticity dominate the chemical bonding [25,33,34,35]. The two most-used indices for quantifying aromaticity are the harmonic oscillator model of aromaticity, based on the geometric structure, and the nucleus-independent chemical shift, based on the magnetic response. Aromaticity is not observable, cannot be directly measured [36], and it is correlated with electronic delocalization [37]. However, in molecular devices, the dynamic structural fluxionality in boron and boron-doped-based molecular systems occur due to electronic delocalization [25,38]. Electronic localization/delocalization contributes significantly to the stability, magnetic properties, and chemical reactivity of a system [36]. Presently, the dynamic structural fluxionality in boron nanoclusters is a topic of interest in nanotechnology [23,39]. The fluxionality of an atomic cluster is highly relevant in terms of its catalytic activity [40], and in boron-based nanoscale rotors, it is a function of the atomic structure, size, bonding, and cluster charge [41]. Doping a boron cluster with metals [3,5,42,43,44,45,46] and non-metals [47] dramatically affects its structure, stability, and reactivity, as a result of the loss in fluxionality of the boron-doped anion B_19_^−^ [48]. It is important to mention, the emission of radiation as a competing cooling channel has to be considered in studying small cationic boron clusters’ stabilities. According to Ferrari et al., this improving agreement between experiment and theory [49]. In this study, we consider that temperature and entropy are critical for elucidating low-energy structures and highlight the importance of understanding the thermal and entropic effects in the Be_6_B_11_^−^ fluxional cluster. In past years, a boron molecular Wankel motor [1,28,50,51,52,53,54] and sub-nanoscale tank treads have been reported [55,56]; however, the entropy and temperature were not considered. In collaboration with the groups of Merino and Zhai, we studied and reported the fluxionality in Be_6_B_11_^−^. The computations indicated that there are two competitive low-energy structures: a helix-type cluster and a fluxional coaxial layered cluster. More recently, another low-energy structure was found in the Be_6_B_11_^−^ cluster by employing a cellular automaton algorithm [57]. However, the putative global minimum-energy structure and its molecular properties strongly depend on the temperature–entropy term [58,59,60,61,62]. In previous works, the barrier energy in a chemical reaction was computed by considering the effect of the temperature–entropy term [63], the temperature-dependent dipole moments were computed for HCl(H_2_O)_n_ clusters [64], the temperature-dependent linear optical properties of the Si(100) surface were computed [65], and more recently, it was considered in a study of gold clusters [66,67,68], and the thermochemical behavior of the sorghum molecule [69]. It is important to bear in mind that experimental studies are conducted in non-zero temperatures and most theoretical density functional studies assume that the temperature is zero and neglect temperature-dependent and entropic contributions; consequently, their finite temperature properties remain unexplored [70,71]. Thus, it is necessary to understand the effect of temperature on cluster properties and the lowest-energy structure’s determination [70,71,72]. Herein, we investigated the effect of the temperature–entropy term on the relative population and its infrared spectra, which as a starting point require the complicated task of search and elucidation of the putative global minimum and its low-energy isomers [58,73,74,75,76]. Considering temperature in Be_6_B_11_^−^ cluster requires deals with thermodynamics of small systems; the Gibbs free energy of classical thermodynamics also applies for small systems, known as the thermodynamics of small systems [77,78,79] or nano-thermodynamics [80]. The thermodynamics of clusters have been studied by various theoretical and simulation tools [58,70,77,81,82,83,84,85,86,87], such as molecular dynamics simulations [6], Monte Carlo, and analytic methods. Under the harmonic superposition approximation, the temperature–entropy term can be computed with the available vibrational frequencies. The entropy effects have been considered for gold, copper, water, and sodium clusters [59,66,67,68,88,89,90,91,92,93]. The spectroscopic properties of the clusters provide insight into their structure and detect structural transformations [94,95,96]. The temperature effects on IR spectra have been studied experimentally and theoretically on small and neutral gold clusters [66,67] and boron clusters [53]. In the same direction, the pristine Au_13_ gold cluster’s thermodynamical stability at finite temperature was studied using the replica-exchange method, which shows a fluxional behavior [88]. Au_N_ (N = 30–147) clusters’ thermodynamics properties were studied employing the Gupta potential and density functional theory (DFT) methodology [68]. The total absorption spectra were computed as the sum of the different spectra in Ag isomers [97]. We employed statistical thermodynamics to compute the Gibbs free energy temperature-dependency and evaluated relative populations’ temperature among the isomers and temperature effects on the IR spectra. We also identified the vibration modes that significantly contribute to the cluster’s zero-point energy, which is strongly dominated at temperatures higher than 377 K, also we find that this isomer has the shortest B–B bond length. Additionally, we investigate the effect of long-range van der Waals interactions (Grimme’s approach, DFT-D3) on solid–solid transformation points. We think that this provides useful information about which isomers will be dominant at hot temperatures. No work has previously been attempted to investigate entropy-driven isomers in the fluxional Be_6_B_11_^−^ cluster to the best of our knowledge. The remainder of the manuscript is organized as follows: Section 2 briefly describes the theory and computational details. Section 3 discusses the lowest-energy structures, energetic ordering at the DFT/Coupled-Cluster Single-Double and perturbative Triple CCSD(T) level of theory, the relative population, and infrared spectroscopy (IR spectroscopy) as a function of entropic-temperature term. Conclusions are given in Section 4.

## 2. Theoretical Methods and Computational Details

### 2.1. Global Minimum Search

Despite advances in computing power, the minimum global search in molecular and atomic clusters remains a complicated task due to several factors. The exploration should be systematic and unbiased [58,98]; a molecule’s degrees of freedom increase with the number of atoms [57,99,100,101,102]; a molecule composed of N atoms possesses 3N degrees of freedom (i.e., The vibrational modes are (3N − 5) for linear molecules and (3N − 6) for nonlinear molecules.); and, as a consequence, the potential/free energy surface depends on a large number of variables. The number of local minima increases exponentially as a function of the number of atoms in the molecule. Moreover, the total energy computation requires a quantum mechanical methodology to produce a realistic energy value. In addition, there should be many initial structures. It is essential to sample a large region of the configuration space to ensure that structures are not missed, resulting in an incomplete sampling of the configuration space, and miscalculation of the thermodynamic properties [73]. A complete sampling of the potential/free energy surface is nearly impossible, but a systematic exploration of the potential energy surface is useful. Although searching for a global minimum in molecular systems is challenging, algorithms dedicated to the search for global minima, such as simulated annealing [103,104,105,106,107,108], the kick method [109,110], genetic algorithms [111,112,113], the gradient embedded genetic algorithm (GEGA) [114,115,116], and basin hopping [117,118], have been designed and applied over the years. In the past few years, one of the authors designed and employed genetic algorithms [9,10,26,119,120] and the kick methodology [64,120,121,122,123,124,125,126] coupled with DFT to explore the potential energy surfaces of atomic and molecular clusters. They led us to solve the minimum global search in a targeted way. In this paper, our computational procedure employs a recently developed and unbiased hybrid strategy as a search global methodology, which combines a modified-kick heuristic and genetic algorithm coupled to DFT that has been implemented in GALGOSON Python code. GALGOSON systematically and efficiently explores the potential/free energy surfaces (PES/FES) of the atomic clusters to find the minimum-energy structure. The methodology consists of a three-step search strategy where in the first and second steps, we explore the PES, and in the third step, we explore the FES. First, the code generates random initial structures with an initial population of 200 individuals per atom in the Be_6_B_11_^−^ cluster using the kick methodology. The process to create 1D, 2D, and 3D structures is similar to that used in previous works [9,57] and is restricted by two conditions [9] that can be summarized as follows: (1) all the atoms are confined inside a sphere with a radius determined by adding all atoms’ covalent radii and multiplied by a factor established by the user, typically 0.9; (2) the bond length between any two atoms is the sum of their covalent radii, modulated by a scale factor established by the user, typically close to 1.0. This allows us to compress/expand the bond length. These conditions avoid the high-energy local minima generated by poorly connected structures (too compact or too loose). Then, structures are optimized at the PBE0/3-21G level of theory by employing Gaussian 09 code. As the second step, all structures lying in the energy range of 20 kcal/mol are re-optimized at the PBE0-GD3/LANL2DZ level of theory and joined with previously reported global minimum structures. Those structures comprise the initial population for the genetic algorithm. The optimization in this stage is at the PBE0-GD3/LANL2DZ level of theory. The criterion to stop the generation is if the lowest-energy structure persists for 10 generations. In the third step, structures at 10 kcal/mol found in the previous step comprise the initial population for the genetic algorithm that uses Gibbs free energy extracted from the local optimizations at the PBE0-D3/def2-TZVP level of theory, considering the zero-point energy (ZPE) corrections. The stop criterion is similar to that used in the previous stage. In the final step, the lowest-energy structures are evaluated at a single-point energy at the CCSD(T)/def2-TZVP//PBE0-D3/def2-TZVP level of theory. All the local optimizations were performed employing the Gaussian 09 code [127].

### 2.2. Thermochemistry Properties

All the information about a quantum system is contained in the wave function; similarly, the partition function provides all the information needed to compute the thermodynamic properties. It indicates the states accessible to the system at temperature T, so the thermodynamic functions are calculated using the temperature-dependent partition function Q shown in Equation (1):

(1) $$Q\left(T\right)=\underset{i}{\sum }g_{i}e^{-{\Delta E}_{i}/k_{B}T}$$

where $g_{i}$ is the degeneracy or multiplicity; using degeneracy numbers is equivalent considering all degenerate states and the sum runs overall energy levels. $k_{B}T$ is the Boltzmann constant, T is the temperature in Kelvin, and ${\Delta E}_{i}$ is the total energy of a molecule [63,128]. An exact calculation of Q could be complicated due to the internal modes’ coupling. A method to decouple the electronic and nuclei modes is through the use of the Born–Oppenheimer Approximation (BOA). This approach states that the electrons move faster than the nuclei and assumes that the molecular wave function is the electronic and nuclear wavefunction product ${\psi =\psi }_{e}\psi_{n}$. The vibrations change the moment of inertia as a consequence, affecting the rotations; this tightly couples the vibrational and rotational degrees of freedom. The separation of rotational and vibrational modes is called the rigid rotor, harmonic oscillator (RRHO) approximation, under this approximation, the molecule is treated rigidly; This is generally good when vibrations are of small amplitude. Here the vibration will be modeled in terms of harmonic oscillator and rotations in the rigid rotor. Within BOA and RRHO approximations, the partition function is factorized into electronic, translational, vibrational, and rotational energies. Consequently, the partition function, q, displayed in Equation (2), can be given as a product of the corresponding contributions [63,129]

(2) $${q=q}_{\mathrm{trans}}q_{\mathrm{rot}}q_{\mathrm{vib}}q_{\mathrm{elec}.}$$

Table 1 shows the contributions of electronic, translational, vibrational, and rotational energies to the canonical partition function.

We computed all the contributions to the partition functions at different temperature T and a standard pressure of 1 atm. The equations shown in Table 1 and Table 2 are equivalent to those given in [63] and any standard text of thermodynamics [128,129], and apply to an ideal gas. The implemented translational partition function in the Gaussian code [127] is the partition function ${q=q}_{\mathrm{trans}}$, given in Table 1. In this study, ${q=q}_{\mathrm{trans}}$ is computed as a function of T and is used to calculate the translational entropy. In addition to using vibrational modes to identify the true lowest-energy structures from transition states, we used them to compute the vibrational partition function. We considered vibrational modes ν under the harmonic oscillator approximation, and the total vibrational energy consists of the sum of the energies of each vibrational mode. In computing the electronic partition, we considered that the energy gap between the first and higher excited states is greater than $k_{B}T$; consequently, the electronic partition function ${q=q}_{\mathrm{elect}}$ is given by $q_{\mathrm{elect}}{=\omega }_{0}$. $q_{\mathrm{rot}}$, $q_{\mathrm{rot}}^{\mathrm{nl}}$, ${q=q}_{\mathrm{elect}}$ and $1{q=q}_{\mathrm{trans}}$ were used to compute the internal energy (U), and entropy (S) contributions given in Table 2.

The vibrational frequencies are calculated employing Gaussian code, and all the information needed to compute the total partition functions is collected from the output Gaussian code. The Gibbs free energy (G) and the enthalpy (H) are computed employing Equations (3) and (4), respectively. In these equations, R is the ideal gas constant and *n* is the amount of substance, and T is the temperature in Kelvin.

(3) H=U+nRT,

(4) G=H−TS.

### 2.3. Boltzmann Population

The properties observed in a molecule are statistical averages over the ensemble of geometrical conformations or isomers accessible to the cluster [130]. Therefore, the molecular properties are ruled by the Boltzmann distributions of isomers, which can change due to the temperature–entropic term [59,64,131], and the soft vibrational modes of clusters make primarily important contributions to the entropy [91]. The relative populations of the low-energy isomers of the Be_6_B_11_^−^ cluster are computed through the probabilities defined in Equation (5)

(5) $$P\left(T\right)=\frac{e^{-{\beta \Delta G}^{K}}}{\sum e^{-{\beta \Delta G}^{K}}},$$

where $\beta =1/k_{B}T$, and $k_{B}$ is the Boltzmann constant, T is the temperature in Kelvin, and ΔG is the Gibbs free energy of the kth isomer. Equation (5) establishes that the molecules will be distributed among energy levels as a function of the energy and temperature. The separation energy among isomers is a critical factor in the computation of the solid–solid transformation temperature, T_ss_, point. T_ss_ occurs when two competing structures are energetically equal, and there is a simultaneous coexistence of structural isomers at T. In other words, the T_ss_ point is a function of the energy difference between two isomers and the relative energy ΔG of the cluster. The Boltzmann distribution finds many applications, such as for native protein structures [132]; for microscopy systems, a temperature T or simulated annealing was applied to the search for minimum-energy structures and rate of chemical reactions [63]. For the calculation of the Gibbs free energies at temperature T, IR spectra at temperature T, and the relative populations, we used a homemade Python/Fortran code called Boltzmann optics full adder (BOFA).

### 2.4. IR Spectra

The vibrational spectra are useful for identifying phases and determining structures [133], among other applications mentioned above. In this study, the IR harmonic spectra for each isomer were calculated employing Gaussian code. All isomers were characterized as minima because we found no negative frequencies in each isomer. The Lorentzian line shape, with a width at half maximum of 20 cm^−1^, was used to compute the IR spectra for each isomer, and the computed vibrations were scaled by a factor of 0.98. The most considerable contribution to total IR spectra is the putative global minimum atomic structure [97], while the isomers located at high energies contribute little to the molecular properties. Therefore, the total IR spectrum is dependent on the temperature results from the contributions of all IR spectra weighted according to their relative populations. In this study, to obtain the total IR spectrum at temperature T, we weighted the IR spectrum of each isomer according to the probabilities computed in Equation (5) and the sum of all of them; thus, we computed the total IR spectrum as a function of the temperature.

### 2.5. Computational Details

We performed the global exploration of the potential and free energy surfaces of the Be_6_B_11_^−^ with a hybrid genetic algorithm GALGOSON written in Python and coupled to DFT. All local geometry optimization and vibrational frequencies were investigated employing DFT as implemented in the Gaussian 09 [127] (Revision D.01) suite of programs; no restrictions in the optimizations were imposed. Final equilibrium geometries and relative energies are reported at the Perdew-Burke-Ernzerhof-hybrid functional (PBE0) [134], and valence triple-zeta polarization basis set of the Ahlrichs group (def2-TZVP) [135], considering the D3 version of Grimme’s dispersion corrections [136] and including the ZPE corrections. (PBE0-D3/def2-TZVP). As Pan et al. [137] reported, the computed relative energies with PBE0 functional are very close to the CCSD(T) values in B_9_^−^ boron cluster. The def2-TZVP basis from the Ahlrichs can improve computations accuracy and describe the Be_6_B_11_^−^ cluster [26]. To gain insight into its energetics, we evaluated the single-point energy CCSD(T)/def2TZVP//PBEO-D3/def2-TZVP level of theory for the putative global minima and the low-energy isomers. The total IR spectra dependent on temperature were computed employing the Boltzmann weighted sum of the IR spectra of each isomer and the relative populations using Boltzmann factors. Both were implemented in BOFA.

## 3. Results and Discussion

### 3.1. The Lowest-Energy Structures and Energetics

**Figure 1:**
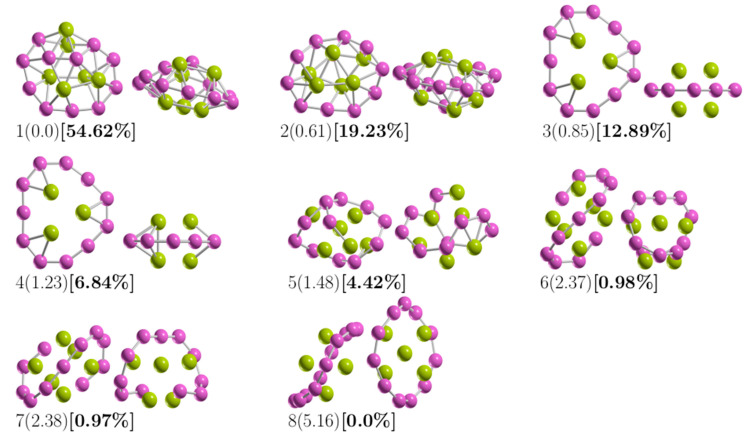
The optimized geometries of the Be_6_B_11_^−^ cluster. The most important energy isomers have two orientations: front and rotated 90 degrees to the plane of the paper. Relative Gibbs free energies in kcal/mol (in round parenthesis) and the relative population (in square brackets) at the PBE0-D3/Def2-TZVP level of theory. The criterion to plot them was until the probability occupation was zero at 298.15 K. The purple- and green-colored spheres represent the boron and beryllium atoms, respectively. (The XYZ cartesian atomic coordinates are available in the Supplementary Information).

Figure 1 shows the lowest-energy structure of Be_6_B_11_^−^ clusters and seven low-energy competing isomers. The criterion for drawing/depicting the structures in Figure 1 was until the percentage of the relative population was zero. The relative Gibbs free energy is given in kcal/mol (round parenthesis) and computed at 298.15 K and 1 atm. In square brackets and bold is the percentage of the relative population computed using Equation (5) at 298.15 K. For the putative global minimum, the optimized average B–B bond length is 1.64 Å. In contrast, the optimized B–Be bond length is 2.01 Å.

**Figure 2:**
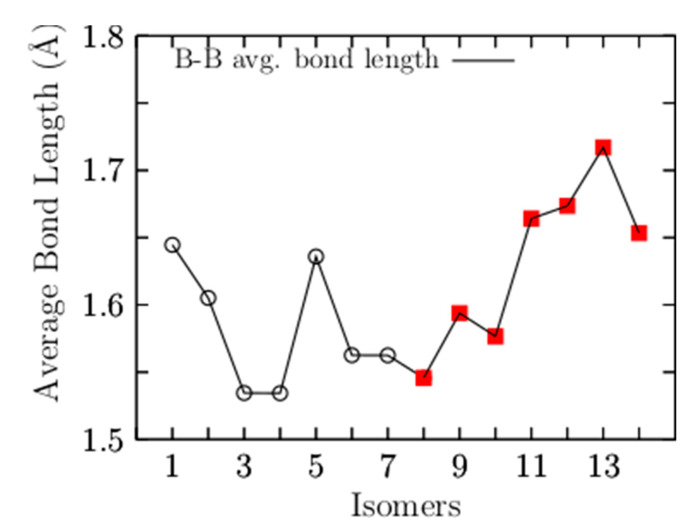
The average B–B bond length as a function of the number of isomers. The isomers are energetically accommodated from the most energetically favorable (1) to the least stable (14). The coaxial triple-layered structures with C_s_ and C_2v_ symmetries, isomers numbers 3 and 4, respectively, have the lowest average bond length of 1.53 Å. The black open circles are the structures with a relative population value different from zero. The filled red squares are isomers with a relative population value of zero. (both of them at 298.15 K).

**Figure A1:**
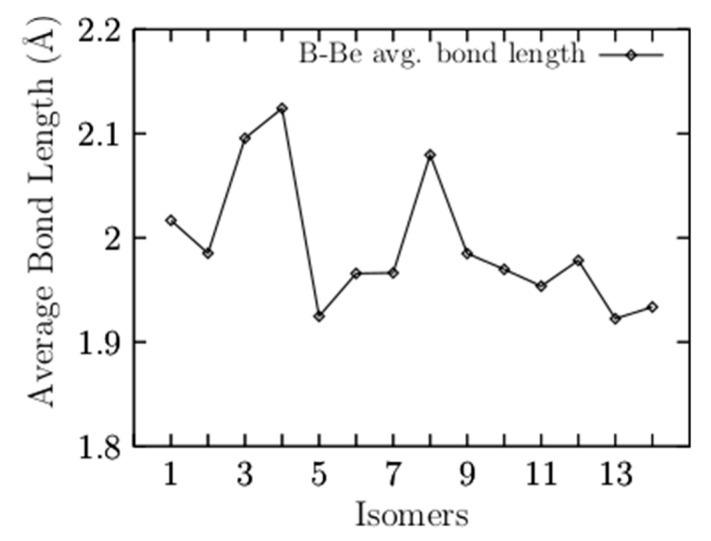
The average Be–B bond length a function of the number of isomers. The isomers are arranged in energy, from the lowest- (1) to the highest-energy structure (14). The coaxial triple-layered structures with C_s_ and C_2v_ symmetries (3 and 4) have the highest average bond lengths of 2.09, and 2.12 Å, respectively.

**Figure A2:**
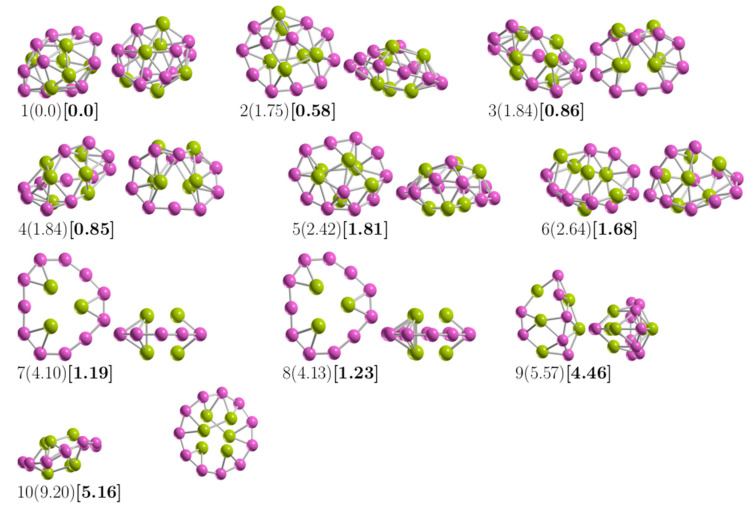
The most important energy isomers shown in two orientations, rotated 90 degrees up to plane paper and front. Single-point relative Coupled-Cluster Single-Double and perturbative Triple (CCSD(T)) energies in kcal/mol (in parentheses) and single-point relative CCSDT energies considering the ZPE energies (in square brackets). Single-point relative CCSD(T) energies agree with those reported previously [57]. The purple- and green-colored spheres represent the boron and beryllium atoms, respectively.

To observe the trend in B–B bond length in the low-energy structures, Figure 2 shows the average bond length for B–B for the 14 lowest-energy isomers energetically accommodated, from the most energetically favorable, isomer number 1, to the least stable, isomer number 14. Our calculations indicated that the largest average value of the B–B bond length is 1.71 Å and belongs to isomer number 13, which is 25 kcal/mol less stable than the putative global minimum. The lowest average value of the B–B bond length is 1.53 Å and corresponds to the isomers coaxial triple-layered structures with C_s_ and C_2v_ symmetries, located at energies of 0.85 and 1.23 kcal/mol above the putative global minima, respectively. The structures are depicted in Figure 1(3,4). In these structures, the lowest average B–B bond length of 1.53 Å is considerably shorter compared with the: (a) length of a typical B–B single bond of 1.72 Å [138], (b) the bond length of the B_8_ and B_9_^−1^ molecular wheels [26,139], and slightly shorter in 2.2% than the B–B double bond length experimentally characterized in the range of 1.57–1.59 Å [140,141]. The average B–B bond length shortens from 1.64 Å to 1.53 Å, suggesting strong hyperconjugation in the coaxial triple-layered structures. The shortening of the B–B bond length is caused by orbital interaction, which is also a cause of C–C bond shortening in the Butyne molecule [142]. Hyperconjugation has been shown in the shortening of B–B and C–C bond lengths [142,143] and which causes increases in the number of electrons shared between regions [142]. Figure A1 (in Appendix A) shows the average bond length for Be–B for the 14 low-energy isomers. The largest average value of the Be–B bond length is 2.0 Å and 2.10 Å, which correspond to the isomer coaxial triple-layered structures with C_s_ and C_2v_ symmetries, respectively. This suggests that if the shortening of the bond length increases the number of electrons shared in that region, the increase in bond length should decrease the number of electrons; consequently, the electron delocalization occurs in the ring of boron atoms. In Figure 1(1), is depicted the putative global minimum with 54% of the relative population, and it has C_1_ symmetry with a singlet electronic state ^1^A. It is a distorted, oblate spheroid with three berylliums in one face and two in the other face. Nine boron and one beryllium atoms form a ring located around the spheroid’s principal axes, and the remaining two boron atoms are located close to the boron ring on one of its faces. The second higher energy structure, at 298.15 K, lies at a Gibbs free energy of only 0.61 kcal/mol above the putative global minima; it has C_1_ symmetry with a singlet electronic state ^1^A. It is a prolate spheroid with 19% of the relative population at a temperature of 298.15 K. The next two higher energy isomers, at 298.15 K, lay 0.85 and 1.23 kcal/mol Gibbs energy above the putative global minimum. They are prolate, coaxial, triple-layered structures with C_s_, and C_2v_ symmetries and singlet electronic states ^1^A and ^1^A_1_, respectively. This clearly shows that the low-symmetry structure C_s_ becomes energetically preferred compared to the C_2v_ symmetry, with a Gibbs free energy difference of 0.38 kcal/mol at 298.15 K due to the entropic effects. This agrees with a similar result found in Au_32_ [98]. According to our computations, those structures are strongly dominant at temperatures higher than 377 K. The next structure shown in Figure 1(5) is located 1.48 kcal/mol above the global minimum; it is close to spherical in shape and corresponds to a prolate structure with C_1_ symmetry and a singlet electronic state ^1^A. This structure makes up only 4.4% of the relative population at 298.15 K. The next two structures, located at a Gibbs free energy of 2.37 kcal/mol above the global minimum, are the chiral–helix-type structures. These were previously reported by Guo et al. [26] as the global minimum and also found with GALGOSON code. They are prolate structures with C_2v_ symmetries and their relative population is around only 1%. We note that the chiral–helix structures are never the lowest-energy structures throughout the entire temperature range. The relative population is zero for structures located at relative Gibbs free energies higher than 5.1 kcal/mol, and at 298.15 K, there is no contribution of these isomers to any total molecular property. A full understanding of the molecular properties requires the search for the global minimum and all its closest low-energy structures [73]. The separation among isomers by energy difference among isomers is an important and critical characteristic that influences the relative population and, consequently, the overall molecular properties. To gain insight into how the energy difference among isomers changes and how the energy ordering of the low-energy structures is affected. We computed the putative global minima and the first seven low-energy structures a single-point energy at the CCSD(T)/def2-TZVP level of theory corrected with the ZPE computed at the PBE0 D3/def2-TZVP level of theory. Figure A2 (in Appendix B) shows the isomers’ energetic ordering considering CCSD(T) energy in kcal/mol in parentheses, and the corrected $\mathrm{CCSD}\left(T\right)+\varepsilon_{\mathrm{ZPE}}$ in kcal/mol in square brackets. At the CCSD(T) level of theory, the global minima, the seven lowest-energy isomers, and the energy order agree with those in a previous work [57], as shown in the first row of Table 3. The second row of Table 3 shows the corrected $\mathrm{CCSD}\left(T\right)+\varepsilon_{\mathrm{ZPE}}$. Interestingly, the energetic ordering of isomers does not change when considering the ZPE.

Nevertheless, the energy difference among isomers were reduced drastically. For example, the energy difference between the first and second isomers was reduced by 66%, from 1.75 to 0.58 kcal/mol; the energy difference between the second and third isomers increased almost 300%, from 0.1 to 0.27 kcal/mol, as shown in rows one and two of Table 1, respectively. This change (increase/decrease) in energy difference among isomers has an enormous impact on the relative population. Consequently, we deduced that the ZPE inclusion is essential to the isomers’ energy ordering and molecular properties. The third row of Table 3 shows the energy order considering the Gibbs free energy computed at 298.15 K; at this temperature, the isomers’ energy ordering changes: the second isomers are the putative global minima, and the first isomers have the fifth-lowest energy. Interestingly, this energy ordering occurs at 298.15 K, and it is a function of the temperature, which we discuss later in the relative population section. The fourth row in Table 3 shows the electronic energy considering the ZPE. It follows the same trend in energy ordering when considering the Gibbs free energy, and it is the same putative global minima. The fifth row in Table 3 details the electronic energy. It almost follows the CCSD(T) energies trend, except isomer number 8 takes second place, located at 0.52 kcal/mol above the putative global minima. The sixth, seventh, and eighth rows in Table 3 show the point-group symmetry, electronic ground state, and the lowest vibrational frequency of each isomer, respectively. When we use the Gibbs free energy to energy order the structures, the second isomers change to first place, becoming the lowest-energy structure; the energy ordering changes drastically, whereas the electronic energy follows a similar trend to that of CCSD(T) energy ordering. This shows us that the level of theory and the inclusion of entropy and temperature change the energy ordering and, therefore, the overall molecular properties.

### 3.2. Relative Population

**Figure 3:**
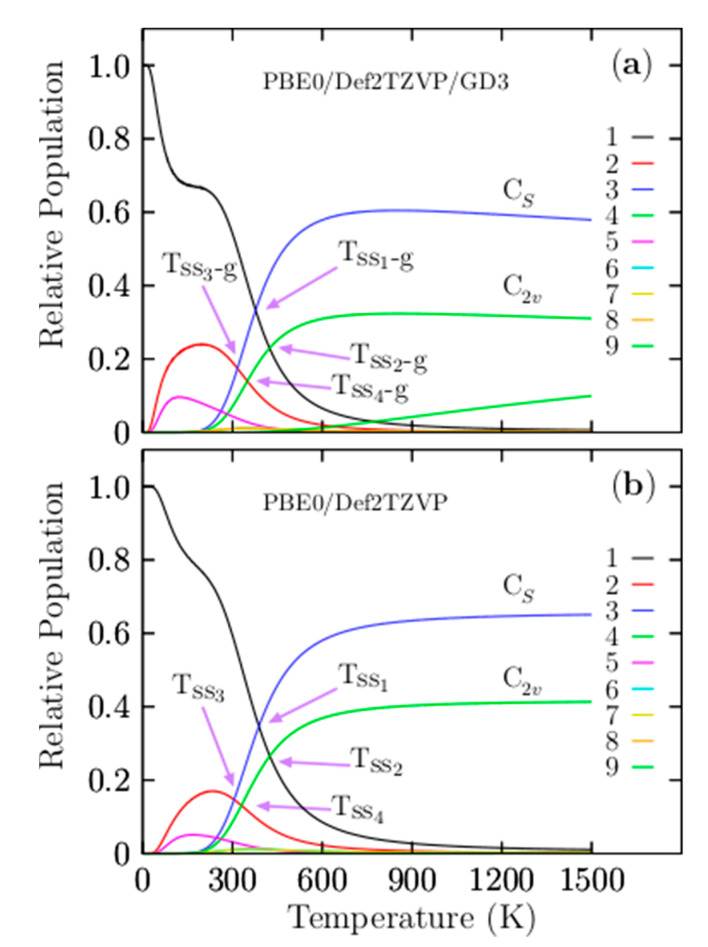
(**a**) The relative population for temperatures ranging from 10 to 1500 K at the PBE0-GD3/def2-TZVP level of theory. (**b**) The relative population (without Grimme’s dispersion (D3)) for temperatures ranging from 10 to 1500 K at the PBE0/def2-TZVP level of theory. Notice that the Grimme’s dispersion’s (D3) effect shifts the solid–solid transition point (T_ss1-g_) to higher temperatures. The low-symmetry C_s_ and C_2v_ coaxial triple-layered structures become strongly dominant at high temperatures.

Figure 3a shows the most important and dominant T_ss1-g_ point located at 377 K with a relative population of 33%. For temperatures ranging from 10 to 377 K, the relative population is strongly dominated by the putative global minima isomer, which is a distorted oblate spheroid with C_1_ symmetry. This relative population is similar to −T^−3^ function, with one point of inflection located at 180 K. Beyond 180 K, it decreases monotonically up to 377 K. At the T_ss1-g_ point, the distorted oblate spheroid with C_1_ symmetry co-exists and competes with the coaxial triple-layered structures with C_s_ symmetry. This implies that the distorted oblate spheroid will be replaced with the coaxial triple-layered structures. Above 377 K, the relative population is strongly dominated by the coaxial triple-layered structures with C_s_ symmetry, located 0.85 kcal/mol above the global minima at 298.15 K. This relative population, depicted by a blue solid line in Figure 3a, behaves as a sigmoid function; from 377 to 600 K, it grows rapidly, and from 600 to 1500 K, it is near-constant with 60%. The second T_ss2-g_ point is located at 424 K, with a relative population of 22.9%; at this point, the global minima distorted oblate spheroid with C_1_ symmetry co-exists and competes with the coaxial triple-layered structures with C_2v_ symmetry, located 1.23 kcal/mol above the global minima at 298.15 K. The relative population of the coaxial triple-layered C_2v_ symmetry depicted with a green-solid line in Figure 3a also behaves as a sigmoid function; up to 600 K; it remains constant, with 32% of the relative population. The T_ss3-g_, and T_ss4-g_ points, displayed in Figure 3a, are located at 316.7 and 349 K axis temperatures with relative populations of 14% and 17%, respectively. These relative populations correspond to the second isomer located at just 0.61 kcal/mol at 298.15 K above the global minima and co-existing at temperatures of 316.7 and 349 K with the coaxial triple-layered structures with C_s_ and C_2v_ symmetries, respectively. In the low-temperature range, this isomer’s relative population, depicted by the red solid line in Figure 3a, is around 20%; up to room temperature, it decreases exponentially to zero. At temperatures of up to 600 K, the relative population is zero; hence, these isomers do not contribute to the molecular properties at high temperatures. A relative population lower than 10%, depicted by the solid purple line, is shown in Figure 3a, corresponding to the isomers located 1.48 kcal/mol above global minima at 298.15 K.

Interestingly, this structure is the putative global minimum when the CCSD(T) energy is employed in the ordering energy. Regardless, this structure’s relative population clearly shows that this structure does not contribute to the molecular properties in all temperature ranges. The average B–B bond length for this structure is 1.63 Å, greatly different to the lowest average B–B bond length of 1.53 Å. This structure has the largest positive contribution to the relative ZPE. This suggests the importance of the global minimum and its closest energy isomers of a potential/free energy surface. Still, the contributions of entropic effects and temperature decide which isomers contribute to the molecular properties in a temperature range of interest. Notably, neither the helix-type structure reported by Guo et al. [26] nor the putative global minimum found in this study, also reported by Yañez et al. [56] at a high level of theory, is the putative global minimum when we consider the entropy. Our results show that the entropic-temperature effect should be considered. One may ask if there is a simple and easy method to elucidate the isomers that provide the largest entopic contributions. This question is to be discussed in the relative ZPE decomposition section.

**Figure 4:**
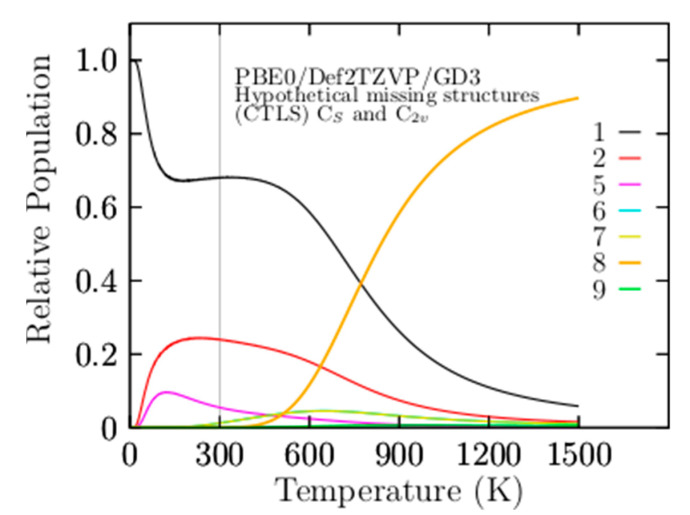
The relative population of the Be_6_B_11_^−^ cluster for the temperatures ranging from 10 to 1500 K, and in the absence of C_2v_ and C_s_ symmetry coaxial triple-layered structures (CTLSs) in a pool of isomers. The distorted coaxial triple-layered structure with C_2v_ symmetry depicted in Figure 1(8) and Figure A2(10) dominates for temperatures higher than 773 K. The lowest-energy structure at T = 0 K does not necessarily have the significant entropic effect. The probability of occurrence of isomer number 9 is depicted in a solid yellow line, and above 733 K strongly dominates. The solid black line is the probability of occurrence of the isomers number one, below 733 K strongly dominate.

**Figure 5:**
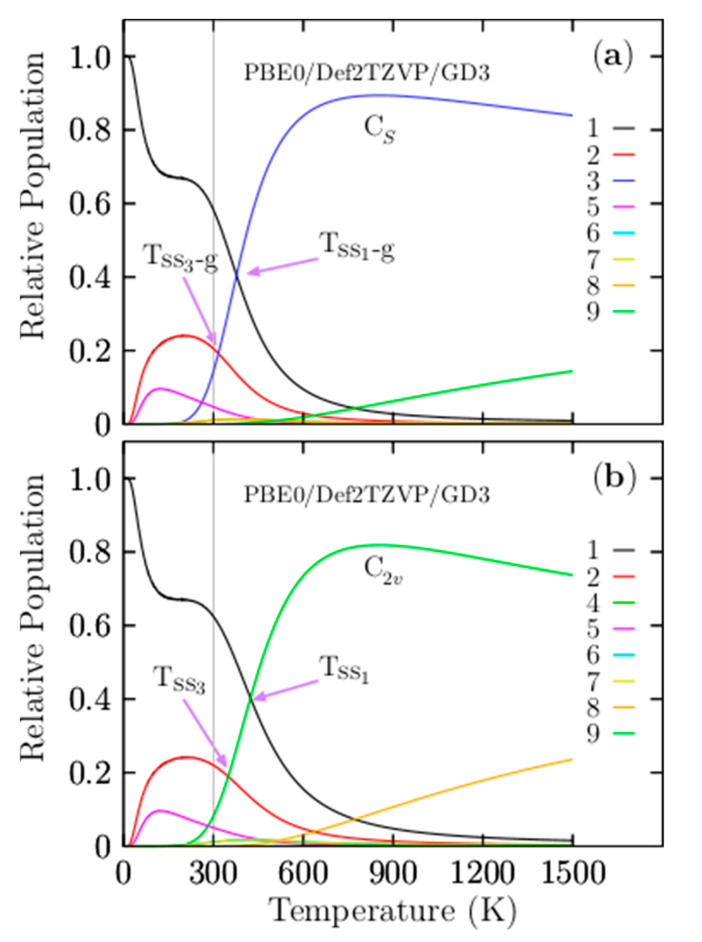
The relative population of the Be_6_B_11_^−^ cluster for temperatures ranging from 10 to 1500 K. (**a**) The relative population of the C_S_ symmetry (blue solid line), in the absence of C_2v_ symmetry coaxial triple-layered structure in a pool of isomers, the low-symmetry C_s_ becomes strongly dominant for temperatures higher than 379 K (T_ss1-g_). (**b**) The relative population of C_2v_ symmetry in the absence of C_S_ symmetry coaxial triple-layered structure in a pool of isomers; the high order-symmetry C_2v_ becomes strongly dominant for temperatures higher than 425 K (T_ss1_) (green line). The absence of isomers with symmetries in the isomers pool database has important consequences for the molecular properties. The shift of the T_ss_ points modifies the IR spectra.

Another question is the effect of Grimme’s dispersion (D3) on the relative population. Figure 3b shows four solid–solid transformation temperature points, T_ss1_, T_ss2_, T_ss3_, and T_ss4_, without Grimme’s dispersion (D3). For ease of numerical of comparison, they are displayed in parentheses in Table 4 together with the probability of occurrence in bold and square brackets. The T_ss1_ and T_ss2_ points shift on the temperature axis to a higher temperature by 10 and 20 K, respectively, whereas the relative population has variations not larger than 1.5%. The T_ss3_, T_ss4_, and T_ss5_ shift on the temperature axis to low temperatures. Initially, this suggests that the dispersion of the relative population indicates a shift of the two dominant T_ss_ points from low to higher temperatures, keeping the relative populations near-constant. In contrast with the T_ss_ points, with lower probability occurrence, T_ss_ points shows a small shift from high to lower temperatures with minimal changes in the relative population. The real properties in a molecule are statistical averages over the ensemble of isomers. Thus, it is crucial to, as far as possible, completely sample the potential energy surface to consider all isomers. The search for low-energy structures is not straightforward, and it could often lead to missing some low-energy isomers. In this respect, we ask what would happen if a low-energy structure was missing when computing the relative populations and the effect on the computation of any molecular properties. Figure 4 shows the computed relative population when the two coaxial triple-layered C_s_ and C_2v_ structures have been removed from the isomers pool database. In the range from 773 to 1500 K, the relative population depicted by the solid yellow line in Figure 4 indicates that the dominant structure is a distorted coaxial triple-layered structure, as depicted in Figure A2(10), located 9.20 kcal/mol above the putative global minimum at the CCSD(T) level of theory. Furthermore, the analysis of the results on the average B–B bond length shown in Figure 2 indicated that the structure with the second-lowest bond length also has the same distorted coaxial triple-layered structure. This result led to a couple of interesting observations in the case of the Be_6_B_11_^−^ cluster. Even at a high level of theory, the lowest-energy structure (at T = 0) does not necessarily have the largest entropic effect. The structure with the lowest B–B bond length is correlated with the largest entropic effect. At 377 to 1500 K in Figure 3a, the relative population depicted by the solid blue line indicates that the coaxial triple-layered structure with C_s_ symmetry is energetically more favorable than the coaxial triple-layered structure with C_2v_ symmetry. These two structures strongly dominate in this temperature range from 377 to 1500 K. These results show that we must consider more than one isomer with point-group symmetries, ranging from low to high symmetry. Figure 5a displays the relative population computed without considering the C_2v_ symmetry coaxial triple-layered structure in the pool database, and panel (b) displays the relative population computed without considering the C_2v_ symmetry coaxial triple-layered symmetry in the pool database. A comparison between the relative populations in Figure 5a,b indicates that the dominant T_ss_ point does not shift when we do not consider the high symmetry structure, and the dominant T_ss_ point shifts from 379 to 425 K when we do not consider the low-symmetry structure. This result led to the observation that it is more important to calculate the relative population considering the low-symmetry structures than only those structures with high symmetries because when we consider low-symmetry structures, the T_ss_ point does not change. In contrast, when we consider only higher-symmetry structures, the T_ss_ changes, with important consequences for the molecular properties when we compute the molecular properties as statistical averages over an ensemble of isomers.

### 3.3. Molecular Dynamics

In this study, to explore and gain insights into the dynamical behavior of Be_6_B_11_^−^ a Born–Oppenheimer molecular dynamics (BOMD) was performed employing the deMon2K program [144] (deMon2k v. 6.01, Cinvestav, Mexico City, Mexico, 2011) at three different temperatures, 1600 K, 2000 K, and 2500 K., and at the PBE/DZVP level of theory. We have chosen the temperatures from 1600 K to 2500 K due to these temperatures are close to the melting points of boron (2349 K) and beryllium (1560 K). The BOMD’s were started from the initial configuration of the coaxial triple-layered structure (the putative global minimum at a temperature of 1500 K), employing a Hoover thermal bath with random initial velocities imposed to the atoms, and for a simulation time of 25 ps with a step size of 1 fs. As the temperature increases, Be_6_B_11_^−^ cluster is subject to dissociation phenomena. Based on the BOMD simulation results, we found the dissociation processes of the Be_6_B_11_^−^ cluster occurs at a temperature of 2000 K, whereas there is no dissociation during the BOMD simulation at 1600 K; the cluster maintains its connectivity at this temperature. At a temperature of 2500 K, the dissociation processes are stronger, and more beryllium atoms are escaped (see the movies in the Supplementary Information). Min Li et al. [145] noted that nanoparticles of tungsten dissociate when the temperature of tungsten nanoparticles is higher than the melting temperature. Our results make sense if we considered that the BOMD of Be_6_B_11_^−^ cluster there is not dissociation at 1600 K, whereas at the temperature of 2000 K, there are dissociation phenomena. From the mentioned previously, we can infer that the melting point of the Be_6_B_11_^−^ cluster is in the temperature ranging from 1600 K to 2000 K.

### 3.4. Contributions of the Vibrational Modes to the ZPE

**Figure 6:**
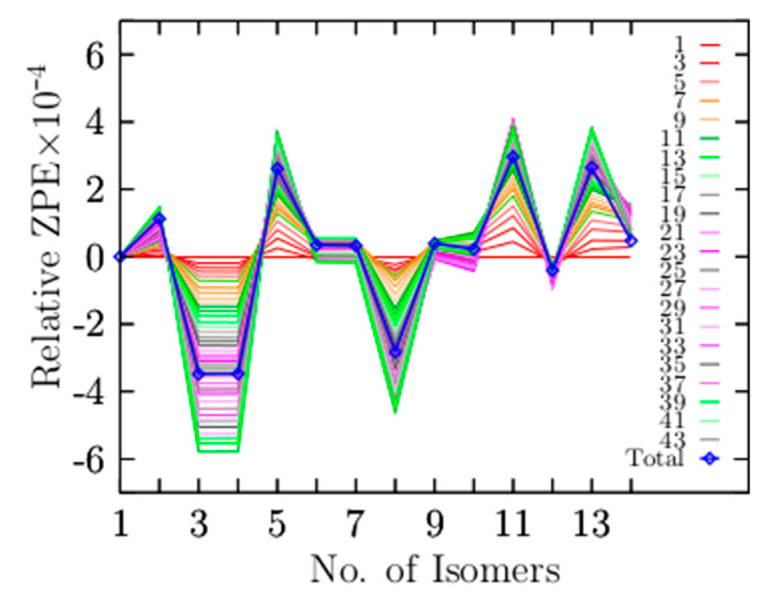
(Color online) Relative zero-point energy (ZPE) decomposition as a function of the vibrational modes (Hartree/particle), with the reference ZPE of the lowest-energy isomer. The x-axis is the number of isomers arranged from the lowest-energy isomer (1) to higher energy isomers (14). The lowest value of the total relative ZPE as a function of the number of isomers is correlated with the isomer that dominates as a global minimum at high temperatures, which corresponds to the coaxial triple-layered structures with C_s_ symmetry. The blue line depicted the total ZPE, taking all 45 vibrational modes of the cluster (3N − 6 modes).

At zero temperature, the lowest-energy structure has electronic energy plus ZPE computed as the sum of all vibration modes. If the system’s temperature increases, the entropic effects start to play an important role, and Gibbs’s free energy determines the lowest-energy structure. Low-vibrational modes significantly contribute to entropy, and it is approximately proportional to the logarithmic sum of low frequencies [146]. High vibrational modes provide small contributions to the vibrational entropy. Equation (6) describes the ZPE, where $\nu_{i}$ is all 3N − 6 vibrational modes of the cluster. Figure 6 shows the relative ZPE as a function of vibrational modes and isomers that are arranged in energy from the lowest- (1) to the highest-energy isomer (14). Remarkably, the smallest value of the total relative ZPE (the minimum ZPE) correlates with the lowest-energy structure at high temperatures. The relative population displayed in Figure 3a shows that isomers three and four, which correspond to the coaxial triple-layered structure with C_s_ and C_2v_ symmetries, respectively, strongly dominate in the temperature range up to 377 K.

(6) $$\mathrm{ZPE}=\frac{1}{2}\sum \nu_{i}$$

Figure 6 shows that isomers three and four, the coaxial triple-layered structures (CTLSs) with C_s_ and C_2v_ symmetries, respectively, have the lowest relative ZPE values. The structures with the lowest relative ZPE are correlated with the structures that strongly dominate the putative global minima at high temperatures. This suggests that those structures possess the highest entropic effects. To understand which of the low-vibrational modes contribute to the lowest ZPE, we decomposed the relative ZPE as a function of the number of modes, adding the number of modes needed to obtain the smallest ZPE value. The blue line in Figure 6 depicts the total relative ZPE employing the 45 vibrational modes. The red solid lines depict the relative ZPE employing the first to the sixth vibrational mode in Equation (6), and so on. The Be_6_B_11_^−^ cluster has 45 vibrational modes; we found that we had to add the lowest 38 vibrational modes to produce the smallest relative ZPE value. The frequency of mode 38 is 1026 cm^−1^, which indicates the highest frequency (cutoff frequency) that contributes to creating the minimum relative ZPE. Therefore, the vibrational frequencies ranging from 46 to 1026 cm^−1^ significantly contribute to entropy. The vibrational mode numbers 39 to 45 (1036–1518 cm^−1^) do not contribute to lowering the relative ZPE, as shown in Figure 6.

### 3.5. Infrared Spectroscopy

In this section, each isomer’s IR spectra and how the relative stabilities contribute to the total IR spectra are discussed. In this study, each isomer’s IR spectra were computed using DFT as implemented in Gaussian 09 code; under the harmonic approximation, anharmonic effects are not considered. The effect of temperature on the total spectra and the total IR spectra were computed as a Boltzmann weighted sum of each isomer’s IR spectra, implemented in BOFA. As the Boltzmann factors depend on temperature, the total resulting IR spectra depend on temperature. In a previous work [64], one of the authors computed the total dipole moment as a dipole moment weighted by the Boltzmann factors and successfully compared it with experimental data. From the experimental point of view, Sieber et al. [97] compared the measured absorption spectrum of the Ag_9_ cluster to a sum of different absorption spectra of the Ag_9_ cluster computed by DFT. Concerning boron clusters, the vibrational spectrum of boron cluster B_13_^+^ was measured by infrared photodissociation spectroscopy and also compared with computed spectra. Experimental spectroscopy studies employing anion photoelectron spectroscopy on boron anions cluster up to B_40_^−^ clusters have been done. Additionally, the structure of neutral boron clusters B_11_, B_16_, and B_17_ was also probed by IR [53].

**Figure 7:**
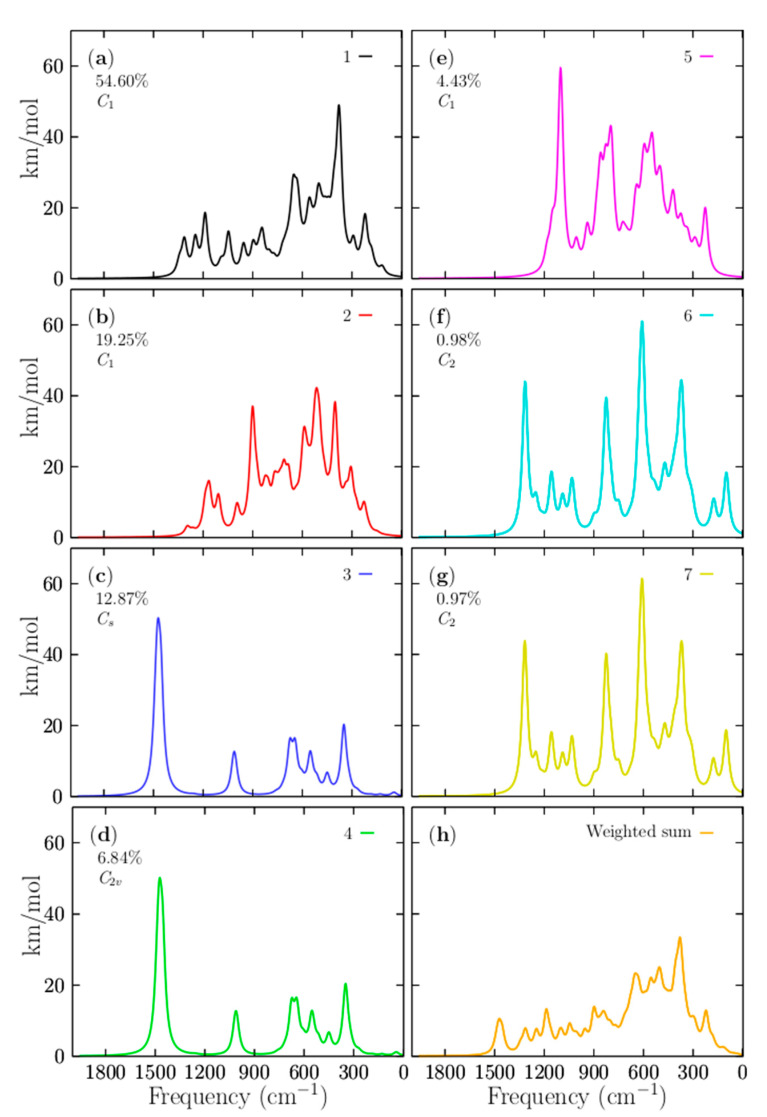
(**a**–**g**) Computed infrared spectra of boron clusters in the range of 1 to 2000 cm^−1^, based on the PBE0 functional with the def2-TZVP basis set, and considering version D3 Grimme’s dispersion (implemented in Gaussian 09 code). (**h**) The weighted IR spectrum Boltzmann sum of the IR spectra’s results of the energetically competing structures, which provide different percentages to the entire IR spectrum. The total weighted IR spectrum shows a peak at 1500 cm^−1^, which is not present in the IR spectrum that belongs to the putative global minimum at 298.15 K shown in (**a**). These IR bands are assigned to the 12.9% contribution of the third isomer, which has coaxial triple-layered structures with C_s_ symmetry located 0.85 kcal/mol above the global minima. The intensities are given in km/mol.

The IR spectrum is related to vibrations that alter the dipole moment. These spectra are usually used to identify functional groups and chemical bond information, and are useful in organic/inorganic chemistry. However, from an experimental perspective, the assignment of IR bands to vibrational molecular modes can be somewhat difficult and requires *ab initio* calculations. In these computations, the temperature is generally not considered, and discrepancies between experimental and calculated IR spectra can result from finite temperature and anharmonic effects. It is also important to remember that the experiments are essential of multi-photon nature, whereas calculations IR spectra assume single-photon processes. Figure 7a–g display the individual IR spectra that belong to the putative global minima and the six lowest-energy structures, respectively, located in the relative energy range up 0 to 2.38 kcal/mol at 298.15 K. Figure 7h shows the Boltzmann weighted spectrum at 298.15 K computed with BOFA. Interestingly, 93% of the total weighted IR spectra was found to be composed of the individual spectral contributions of the four lowest-energy structures located at an energy scale-up of 0 to 1.23 kcal/mol. The other 7% of the Boltzmann weighted spectra is composed of the IR spectra of the three structures located in the energy range from 1.48 to 2.38 kcal/mol. In the total weighted Boltzmann IR spectrum in Figure 7h, there are three segments on its frequency axis. The first segment is located in the frequency range of 0 to 700 cm^−1^. The main bands observed in this range correspond to the IR vibrational modes of the global minimum. The highest peak is located in the 387 cm^−1^ frequency axis, which corresponds to compression of the main ring formed by 10 boron atoms. It is located mainly on one side of the ring, accompanied by the vibrations of the two beryllium atoms. The second band is located at 669 cm^−1^ in the frequency axis. This corresponds mainly to the 10-ring boron’s small asymmetric vibration and a minor vibration of the six beryllium atoms. The third peak is located at 225 cm^−1^ on the frequency axis. It corresponds mainly to a stretching of the boron atom that does not form part of the boron ring, together with the two beryllium atoms located close to the boron. The second segment is located in the frequency range of 700 to 1400 cm^−1^. In the Boltzmann weighted IR displayed in Figure 7h, the band observed at 900 cm^−1^ is mainly composed of the 19.2% contribution of the individual IR spectrum of the second isomer that lay 0.61 kcal/mol above the global minimum; this vibrational mode of the second isomer corresponds to the stretching of the three beryllium atoms located on one side along with a boron atom, together with the stretching of one of the boron atoms. The band observed at 1200 cm^−1^ (Figure 7h) is mainly associated with the global minima’s IR spectrum, which corresponds to the boron atoms’ unique stretching. There is almost no vibration of the beryllium atoms. The band observed at 1500 cm^−1^ (Figure 7h) is completely composed of the contribution of 12% of the individual IR spectra of the third isomer, which has a coaxial triple-layered structure with C_s_ symmetry located 0.85 kcal/mol above the putative global minima. The fourth isomer’s contribution is the coaxial triple-layered structure with C_2v_ symmetry located 1.23 kcal/mol above the global minimum. The different symmetries of the CTLSs (C_2v_ and C_s_) are responsible for the different contributions to the total weighted IR spectrum. The low-symmetry isomers become more stable at high temperatures as a result of entropic effects. Interestingly, neither individual IR spectrum of the putative global minimum nor the individual IR spectrum of the second isomer, which was 0.61 kcal/mol above the putative global minimum, has any IR band in the range of 1500 cm^−1^. Based on this, we assigned this band at 1500 cm^−1^ in the total Boltzmann weighted IR spectrum to the third and four isomers, which have a coaxial triple-layered structure with two different symmetries. The helix-type structures proposed by Guo et al. [26] have a small contribution to the IR spectra in all ranges of temperature. The methodology employed in this paper for the assignment of the IR bands demonstrates that the total IR spectra are a mixture of many contributions from the low-energy structures. In this cluster, the total IR spectrum’s low-energy region is attributed to the putative minimum global contribution. In contrast, the high-energy region of the total IR spectrum is attributed to the isomers’ contribution on the high-energy axis.

**Figure 8:**
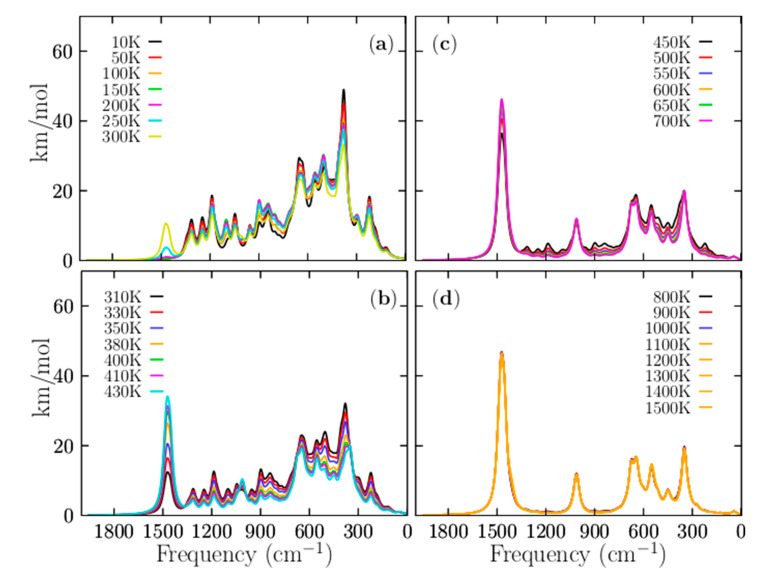
(Color online) The temperature-dependent weighted infrared spectrum of the Be_6_B_11_^−^ cluster, computed at the PBE0-D3/def2-TZVP level of theory from temperature ranging 10 to 1500 K. The temperature effects are considered through Boltzmann factors at (**a**) 10–300, (**b**) 310–430, (**c**) 450–700, and (**d**) 800–1500 K. The most considerable change in the spectrum occurs around the dominant T_SS1-g_ point, (377 K) where there are at least two energetically competing structures. From 450 to 1500 K, the weighted IR spectrum is dominated by the coaxial triple-layered structures.

Figure 8 displays the IR spectra computed as a function of temperature. Figure 8a shows the total Boltzmann weighted IR spectra in the temperature range of 10 to 300 K. Please note that the IR spectrum at low temperatures is strongly dominated by the individual IR spectrum of the putative global minimum; this finding agrees with the relative population displayed in Figure 3. Below 377 K, the relative population is strongly dominated by the putative global minimum. The band observed at 1500 cm^−1^ in Figure 8a starts to increase at 200 K (pink line), increases again at 250 K (cyan line), and increases further at 300 K (yellow line). This IR band has contributions from the individual IR spectra of the CTLSs with C_s_ and C_2v_ symmetries. It is in complete agreement with the relative population displayed in Figure 3a. The relative population of the CTLSs start to increase at 200 K. Figure 8b shows the IR spectra in the range of 310 to 410 K. Within this temperature range, most solid–solid transitions occur with different probabilities of occurrences as shown in Figure 3a; therefore, large changes in the total weighted IR spectra are also expected. In Figure 8b, the IR band located at 1500 cm^−1^ continues increasing at 310 K, and it persists, increasing to 430 K (cyan line). This vibrational mode pertains to an individual IR spectrum of the isomer with CTLSs displayed in Figure 7c. This is completely in agreement with the relative population displayed in Figure 3a. From 377 to 1500 K, the relative population is strongly dominated by the coaxial contributions of the triple-layered structures with C_s_ and C_2v_ symmetries. The appearance and constant growth of the peak located at 1500 m^−1^ in the weighted total IR spectrum displayed in Figure 8b, as a function of temperature, indicate the coexistence and competition of at least two strongly dominant structures at a specific finite temperature (377 K). Most importantly, the constant growth of the peak located at 1500 cm^−1^ is indicative that putative global minimum interchange occurs as a function of temperature. This suggests that we must search exhaustively and systematically for the putative global minimum on the potential/free energy surface and its full distribution of all low-energy structures if we want to assign IR bands to specific vibrational modes. This paper shows how some IR bands in the Boltzmann weighted total IR spectrum belong to the IR spectra of isomers located on the higher energy axis. In summary, in the Boltzmann weighted total IR spectrum shown in Figure 8b, the low-frequency range is dominated by the contributions of the putative global minimum, whereas the high-frequency range is dominated by geometric structures located at higher energies. Figure 8b shows the IR spectra in the range of 310 to 410 K. Within this temperature range, most solid–solid transitions occur with different probabilities, as shown in Figure 3a; therefore, large changes in the total weighted IR spectra are also expected. In Figure 8b, the IR band located at 1500 cm^−1^ continues increasing at 310 K, and it persists, increasing up to 430 K (cyan line). This vibrational mode pertains to an individual IR spectrum of the coaxial triple-layered isomer displayed in Figure 7c. This is completely in agreement with the relative population displayed in Figure 3a. The peak located at 1500 cm^−1^, shows in Figure 8, panels (a–d), is present only in temperatures higher than 300 K, where the coaxial triple-layer structures start to be the lowest-energy structures. The above-mentioned reasons indicate that the vibrational modes located in the range of 1036 to 1518 cm^−1^ are responsible for the cluster’s fluxionality. The B−B stretching modes of the B_11_ ring are located in the range of 1036 to 1518 cm^−1^. This is correlated with hyperconjugation, delocalization, and fluxionality of the cluster.

## 4. Conclusions

In summary, we systematically explored the potential and free energy surface of the Be_6_B_11_^−^ cluster using an unbiased hybrid, efficient, and multistep/multilevel algorithm implemented in *Python* and coupled to DFT. The temperature effects were considered employing Gibb’s free energy. If the system’s temperature is increased, entropic effects start to play an important role, and Gibbs’s free energy determines the lowest-energy structure.

We computed the relative population as a function of temperature using Boltzmann factors and the IR spectra dependent on temperature as a Boltzmann weighted sum of each IR spectrum’s isomer. Here, we demonstrate that the temperature and entropic effects produce several competing structures, so a mixture of isomers co-exist at a specific temperature. Our computations showed (with relative population) that the low-symmetry isomers have a higher stability than isomers with high symmetry at high temperatures as a result of the entropic effect. The CTLSs with C_s_ symmetry are the putative global minima above 377 to 1500 K due to entropic effects. There are four T_ss_ points in the relative population of the Be_6_B_11_^−^ cluster; the most important and dominant of these is the T_ss_ point located at 377 K with a relative population of 33%. Additionally, our results give insight into the long-range van der Waals interactions effects on the solid–solid transformation temperature points, hence the molecular properties. Indeed, the effect of dispersion shifts up in temperature the dominant T_ss_ point, keeping the relative population almost invariant. The other T_ss_ points shifted down on the temperature axis, so there is no clear trend in the up/downshifts in the Be_6_B_11_^−^ cluster. Remarkably, the CTLSs with C_s_ and C_2v_ symmetries have the lowest B–B bond length, and the same geometrical structures have the lowest relative ZPE. This suggests that both trends shortening of the B–B bond length and lowest relative ZPE are correlated with entropic effects. Analysis of our results leads to an interesting observation: The strong dominant putative global minimum, under high-temperature conditions, has the shortest B–B bond length and the lowest relative ZPE. The low-vibrational modes significantly contribute to entropy, whereas high vibrational modes provide small contributions to entropy. The Be_6_B_11_^−^ cluster has 45 vibrational modes. We found the range of frequencies—the lowest to the highest vibrational modes that contribute to ZPE by computing the ZPE as a function of vibrational modes. We needed to sum the first 38 modes that contribute to zero-point energies; the frequency range was between 46 and 1026 cm^−1^. Vibrational modes outside of this range do not contribute to the ZPE. At the energy single-point CCSD(T) level of theory, the energetic ordering of isomers changes with respect to employing the electronic or Gibb’s free energies. The inclusion of the ZPE in CCSD(T) energies illustrates that the energy difference among isomers reduces drastically, which suggests that the dominant putative global minimum at zero temperature when we employ the CCSD(T) energies will change with the inclusion of temperature.

The properties observed in a molecule are statistical averages over the ensemble of geometrical conformations or isomers accessible to the cluster, so the molecular properties are ruled by the Boltzmann distributions of isomers, which can change significantly with temperature, primarily due to entropic effects. We computed the IR spectra dependent on temperature as a Boltzmann weighted sum of each IR spectrum’s isomer. Our computations showed that the competing structures provide a different percentage to the entire molecular properties and IR spectra, in detail, the molecular properties cannot be attributed to only the lowest-energy structure. The structures located at high energy above the putative global minimum that have a significant energy difference among isomers on the potential/free energy surface do not contribute to the entire IR spectrum. Despite the number of isomers growing exponentially, the main contribution to the molecular properties comes from the low-energy structures close to the global minimum where the weighted Boltzmann factors’ temperature dependence are different from zero. (This depends strongly on the energy separation; if the energy separation is significant, the IR spectrum does not change.) The spectra that belong to the low-energy structure dominate the IR spectrum of the Be_6_B_11_^−^ cluster at low-temperature structures, whereas at high temperatures, it is strongly dominated by the spectra of structures located at high energy above the putative lowest-energy structures. The increase/decrease in a peak/band in the IR spectra as a function of temperature is a clear signature of an interchange of the dominant lowest-energy structure. With the IR spectra, we illustrated that the main contributions to the molecular properties are from the low-energy structures that are very close to the global minimum where the weighted Boltzmann factors’ temperature dependence is different from zero.

The present study highlights the importance of entropy-temperature effects and what happens when some low-energy structures are not considered. We show that symmetry plays an important role in the definition of the global minimum and hence in molecular properties. We demonstrate that dispersion effects have a little change to the T_ss_ points in temperature scale. All of these effects have an impact on the spectroscopic and any other property of a molecular system. The Boltzmann-IR-spectra as a function of temperature were presented. The spectra unravel that the peak located at 1500 cm^−1^, shows in Figure 8, panels (a–d), is present only in temperatures higher than 300 K, where the CTLSs start to be the lowest-energy structures. The above-mentioned reasons indicate that the vibrational modes located in the range of 1036 to 1518 cm^−1^ are responsible for the cluster’s fluxionality.

An immediate future project is the computation of the optical spectra and other molecular properties employing the methodology described in this study and the computation of the relative population in many atomic and molecular clusters of interest employing higher levels of theory.

## Figures and Tables

**Table 1:** Contributions to the partition function.

Contributions	Partition Function
Translational	$q_{\mathrm{trans}}={\left(\frac{2{\pi \mathrm{mk}}_{B}T}{h^{2}}\right)}^{\frac{3}{2}}\frac{k_{B}T}{P}$
Rotational linear	$q_{\mathrm{rot}}^{l}=\frac{T}{{\sigma \Theta }_{\mathrm{rot}}}{,\;\Theta }_{\mathrm{rot}}=\frac{\hbar^{2}}{2{\mathrm{Ik}}_{B}}$
Rotational nonlinear	$q_{\mathrm{rot}}^{\mathrm{nl}}=\frac{\pi^{1/2}}{\sigma }\left[\frac{T^{3/2}}{{\left(\Theta_{\mathrm{rotA}}\Theta_{\mathrm{rotB}}\Theta_{\mathrm{rotC}}\right)}^{1/2}}\right]{,\;\Theta }_{\mathrm{rotj}}=\frac{\hbar^{2}}{2I_{j}k_{B}},\;j=A,B,C$
Vibrational	$q_{\mathrm{vib}}^{\mathrm{pol}}=\overset{n_{{ʋ}^{\left[a\right]}}}{\underset{i=1}{\prod }}\frac{e^{-\Theta_{{\mathrm{vib}}_{i}}/2T}}{1-e^{-\Theta_{{\mathrm{vib}}_{i}}/T}}{,\;\Theta }_{{\mathrm{vib}}_{i}}=\frac{{h\nu }_{i}}{k_{B}}$
Electronic	$q_{\mathrm{elec}}{=\omega }_{0}$

**Table 2:** Contributions to internal energy and enthalpy.

Contributions	Internal Energy	Entropy
Translational	$U_{\mathrm{trans}}=\frac{3}{2}\mathrm{RT}$	$S_{\mathrm{trans}}=R\left({\ln\;q}_{\mathrm{trans}}+\frac{5}{2}\right)$
Rotational linear	$U_{\mathrm{rot}}^{l}=\mathrm{RT}$	$S_{\mathrm{rot}}^{l}=R\left({\ln\;q}_{\mathrm{rot}}^{l}+1\right)$
Rotational nonlinear	$U_{\mathrm{rot}}^{\mathrm{nl}}=\frac{3}{2}\mathrm{RT}$	$S_{\mathrm{rot}}^{\mathrm{nl}}=R\left({\ln\;q}_{\mathrm{rot}}^{\mathrm{nl}}+\frac{3}{2}\right)$
Vibrational	$U_{\mathrm{vib}}^{\mathrm{pol}}=R\overset{n_{{ʋ}^{\left[a\right]}}}{\underset{i}{\sum }}\Theta_{{\mathrm{vib}}_{i}}\left(\frac{1}{2}+\frac{1}{e^{\Theta_{{\mathrm{vib}}_{i}}/T}-1}\right)$	$S_{\mathrm{vib}}^{\mathrm{pol}}=R\overset{n_{ʋ}}{\underset{i}{\sum }}\left[\frac{\Theta_{{\mathrm{vib}}_{i}}/T}{e^{\Theta_{{\mathrm{vib}}_{i}}/T}-1}-\ln\left(1-e^{-\Theta_{{\mathrm{vib}}_{i}}/T}\right)\right]$
-	$\Theta_{{\mathrm{vib}}_{i}}=\frac{{h\nu }_{i}}{k_{B}}$	
Electronic	$U_{\mathrm{elec}}=0$	$S_{\mathrm{elec}}{=R\;\ln\;q}_{\mathrm{elec}}$

**Table 3:** The relative energies in kcal·mol^−1^, coupled-cluster single-double and perturbative triple CCSD(T), CCSD(T) with zero-point energy ($\varepsilon_{\mathrm{ZPE}}$), ($\mathrm{CCSD}\left(T\right)+\varepsilon_{\mathrm{ZPE}}$), and Gibbs free energy (ΔG) at 298.15 K, electronic energy ($\varepsilon_{0}$), electronic energy with $\varepsilon_{\mathrm{ZPE}}$ ($\varepsilon_{0}+\varepsilon_{\mathrm{ZPE}}$), point-group symmetry, electronic ground state, and the lowest frequency in cm^−1^ for eight low-energy isomers. (Two isomers need to raise the frequencies below 100 cm^−1^ to 100 cm^−1^ to avoid divergence errors).

Be_6_B_11_^−^	Level	i_1_	i_2_	i_3_	i_4_	i_5_	i_6_	i_6_	i_8_
	CCSD(T)	0.0	1.75	1.84	1.84	4.10	4.13	2.64	2.42
	$\mathrm{CCSD}\left(T\right)+\varepsilon_{\mathrm{ZPE}}$	0.0	0.58	0.85	0.86	1.19	1.23	1.68	1.81
	ΔG	0.0	−1.48	0.89	0.88	−0.63	−0.25	4.14	−0.87
Be_6_B_11_^−^	$\varepsilon_{0}+\varepsilon_{\mathrm{ZPE}}$	0.0	−0.29	1.51	1.52	2.41	2.42	5.0	−0.08
	$\varepsilon_{0}$	0.0	0.87	2.50	2.50	5.32	5.32	5.96	0.52
	Point-Group Symmetry	C_1_	C_1_	C_2_	C_2_	C_S_	C_2v_	C_1_	C_1_
	Electronic ground state	^1^A	^1^A	^1^A	^1^A	^1^A′	^1^A_1_	^1^A	^1^A
	Frequencies (cm^−1^)	230	119	102	100	46	43	161	151

**Table 4:** For ease of comparison, the five points T_ss_ at the PBE0-D3/def2-TZVP level considering Grimme’s dispersion (D3), (T_ssi-g_) and at the PBE0/def2-TZVP (T_ssi_) level of theory without dispersion are shown. T_ss_ points are displayed in parentheses together with the probability of occurrence at that point in bold and parentheses.

$T_{{\mathrm{SS}}_{i}-g}/T_{{\mathrm{SS}}_{i}}$	PBE0-D3/def2-TZVP	PBE0/def2-TZVP
1	(377)/(33)	(388)/(34.5)
2	(424)/(22.9)	(444)/(22.8)
3	(316.7)/(14)	(305.4)/(14.7)
4	(349)/(17.6)	(346.6)/(12.2)
5	(258)/(5.7)	(246)/(4.2)

## Data Availability

The movies of molecular dynamics and atomic coordinates presented in this study are openly available in https://www.mdpi.com/1996-1944/14/1/112/s1.

## Supplementary Materials

The following are available online at https://www.mdpi.com/1996-1944/14/1/112/s1, All XYZ atomic coordinates optimized of the Be_6_B_11_^−^ cluster at the PBE0-D3/def2-TZVP/Freq. (S1). Movies of the molecular dynamics simulation of Be_6_B_11_^−^ cluster at 1600 K, 2000 K, and 2500 K are shown in the supplementary material, MD_Be6B11_T_1600.mp4, MD_Be6B11_T_2000.mp4, MD_Be6B11_T_2500.mp4, respectively. The Boltzmann Optics Full Adder (BOFA) *Python* code supporting the findings of this study is available from the corresponding author upon reasonable request.

## Abbreviations

The following abbreviations are used in this manuscript:

DFT	Density Functional Theory
CCSD(T)	Coupled-cluster single-double and perturbative triple
ZPE	Zero point energy
