# Supplementary material for: Exploration of Free Energy Surface and Thermal Effects on Relative Population and Infrared Spectrum of the Be6B11− Fluxional Cluster
Source: Materials (Basel). 2020 Dec 29;14(1):112. doi: 10.3390/ma14010112 (PMC7796227; doi:10.3390/ma14010112)
Supplement: Supplementary file 1 [file materials-14-00112-s001.zip › figures_and_Supplementary_Be6B11/Supplementary Information.pdf]

materials

Supplementary information

Exploration of Free Energy Surface and Thermal Effects on Relative Population and Infrared Spectrum of the $Be_6B_{11}^-$ Fluxional Cluster

The XYZ cartesian atomic coordinates $Be_6B_{11}^-$

```
17
0.000000000             Be6B11_0000.out
Be    0.871471000000    −2.240509000000    0.377306000000
Be    −1.189569000000   0.091524000000     −1.070090000000
Be    −1.553783000000   −0.035182000000    1.029073000000
Be    0.353918000000    0.927628000000     1.191148000000
B     −2.388865000000   −1.286223000000    −0.191009000000
Be    0.902109000000    0.576938000000     −1.026034000000
B     2.138354000000    1.405375000000     0.186226000000
B     2.104451000000    −1.290639000000    −0.677320000000
B     −0.695071000000   2.012979000000     −0.103576000000
B     −2.937178000000   0.167009000000     −0.293782000000
B     −0.966176000000   −1.813381000000    0.224983000000
B     0.854208000000    2.267959000000     0.001131000000
B     1.541700000000    −0.519470000000    0.705420000000
B     −2.190898000000   1.507723000000     −0.161535000000
B     2.776437000000    0.070546000000     −0.362169000000
Be    0.251303000000    −1.389407000000    −1.254458000000
B     0.054679000000    −0.866672000000    1.274075000000
17     0.617460000             Be6B11_0001.out
Be    −1.462704000000   −0.014249000000    0.967655000000
B     2.728694000000    0.030013000000     −0.619010000000
B     −2.146695000000   −1.259141000000    −0.336682000000
B     2.196858000000    1.375491000000     0.031415000000
B     −0.880491000000   −1.823626000000    0.448212000000
B     1.934511000000    −1.299414000000    −0.736505000000
B     −0.694414000000   1.931806000000     0.025551000000
Be    0.874964000000    0.501056000000     −0.992441000000
B     −2.883951000000   0.123283000000     −0.317897000000
Be    1.826138000000    −0.480368000000    0.902917000000
Be    0.408897000000    0.951482000000     1.344252000000
B     0.772439000000    −1.870483000000    0.277861000000
```

B	−2.220982000000	1.503653000000	−0.070210000000
Be	−0.113249000000	−1.338601000000	−1.218981000000
B	0.106145000000	−0.876434000000	1.446090000000
B	0.872097000000	2.199862000000	0.022620000000
Be	−1.264310000000	0.336919000000	−1.217707000000

17

0.855910000 Be6B11_0002.out

Be	−0.597631000000	1.124894000000	1.052561000000
Be	−0.597631000000	1.124894000000	−1.052561000000
B	−2.238140000000	−1.547111000000	0.000000000000
B	1.559153000000	1.828212000000	0.000000000000
Be	−0.597631000000	−1.125067000000	1.051152000000
B	0.289604000000	2.707478000000	0.000000000000
B	−2.209386000000	0.001996000000	0.000000000000
B	−1.233725000000	2.689543000000	0.000000000000
B	−2.233185000000	1.551289000000	0.000000000000
Be	1.279363000000	0.001246000000	−1.086218000000
Be	1.279363000000	0.001246000000	1.086218000000
B	−1.242591000000	−2.688862000000	0.000000000000
Be	−0.597631000000	−1.125067000000	−1.051152000000
B	2.675935000000	0.757041000000	0.000000000000
B	0.280383000000	−2.711048000000	0.000000000000
B	2.670570000000	−0.762843000000	0.000000000000
B	1.546820000000	−1.827411000000	0.000000000000

17

1.230527500 Be6B11_0003.out

materials

MDPI

B	0.000000000000	1.549129000000	2.235698000000
B	0.000000000000	1.827820000000	−1.553909000000
B	0.000000000000	−0.759893000000	−2.673713000000
B	0.000000000000	−2.707671000000	−0.285066000000
B	0.000000000000	−1.549129000000	2.235698000000
B	0.000000000000	−1.827820000000	−1.553909000000
B	0.000000000000	0.759893000000	−2.673713000000
B	0.000000000000	2.707671000000	−0.285066000000
B	0.000000000000	2.688931000000	1.237960000000
B	0.000000000000	0.000000000000	2.207900000000
B	0.000000000000	−2.688931000000	1.237960000000
Be	1.052108000000	1.124714000000	0.598858000000
Be	1.085258000000	0.000000000000	−1.278866000000
Be	1.052108000000	−1.124714000000	0.598858000000
Be	−1.052108000000	1.124714000000	0.598858000000
Be	−1.085258000000	0.000000000000	−1.278866000000
Be	−1.052108000000	−1.124714000000	0.598858000000
17			
1.487802500	Be6B11_0004.out		
B	−0.809816000000	1.579400000000	0.643022000000
Be	0.596573000000	−0.814845000000	2.010115000000
B	0.051091000000	1.760054000000	−0.791777000000
B	1.580974000000	1.309936000000	−0.966076000000
B	−2.070825000000	0.461623000000	0.890950000000
B	−0.603948000000	0.506270000000	1.857829000000
B	2.628206000000	0.531768000000	−0.066162000000
Be	0.883805000000	0.850245000000	0.737351000000
Be	−0.903703000000	−1.025277000000	0.582096000000
B	−2.628548000000	−0.531967000000	−0.178140000000
B	−1.569501000000	−1.073309000000	−1.210345000000
B	−0.048006000000	−1.595631000000	−1.037654000000
Be	1.608400000000	−0.779034000000	−1.006380000000
Be	0.006693000000	0.158977000000	−1.774212000000
Be	−1.600348000000	0.919041000000	−0.888479000000
B	2.151414000000	−0.709603000000	0.737654000000
B	0.845823000000	−1.685827000000	0.392307000000
17			
2.377597500	Be6B11_0005.out		
B	−1.398792000000	−2.376827000000	−0.608428000000
B	1.860269000000	1.944117000000	0.832486000000

B	0.000000000000	−2.314681000000	−1.284177000000
B	1.398792000000	2.376827000000	−0.608428000000
B	−1.860269000000	−1.944117000000	0.832486000000
B	1.198910000000	1.039826000000	1.873576000000
Be	−0.846279000000	−0.686255000000	−1.602453000000
B	0.000000000000	2.314681000000	−1.284177000000
Be	0.026437000000	−1.647024000000	0.486714000000
Be	1.524481000000	0.000032000000	0.331634000000
B	0.978975000000	−1.100162000000	−1.127179000000
Be	−0.026437000000	1.647024000000	0.486714000000
B	−1.198910000000	−1.039826000000	1.873576000000
B	−0.978975000000	1.100162000000	−1.127179000000
Be	−1.524481000000	−0.000032000000	0.331634000000
Be	0.846279000000	0.686255000000	−1.602453000000
B	0.000000000000	0.000000000000	1.882013000000
17			
2.383245000	Be6B11_0006.out		
Be	0.000000000000	1.524670000000	0.325548000000
Be	0.000000000000	−1.524670000000	0.325548000000
Be	−1.646433000000	0.020113000000	0.488496000000
Be	1.646433000000	−0.020113000000	0.488496000000
B	1.104502000000	−0.972620000000	−1.129209000000
B	−1.104502000000	0.972620000000	−1.129209000000
B	0.000000000000	0.000000000000	1.882165000000
Be	−0.681645000000	−0.847320000000	−1.608316000000
Be	0.681645000000	0.847320000000	−1.608316000000
B	1.033017000000	1.204123000000	1.875487000000
B	−1.033017000000	−1.204123000000	1.875487000000
B	−2.314461000000	−0.012187000000	−1.282308000000
B	2.314461000000	0.012187000000	−1.282308000000
B	−1.936501000000	−1.869964000000	0.837138000000
B	1.936501000000	1.869964000000	0.837138000000
B	2.363598000000	1.412169000000	−0.606771000000
B	−2.363598000000	−1.412169000000	−0.606771000000
17			
5.164952500	Be6B11_0007.out		
Be	−1.140416000000	0.103343000000	1.097260000000
Be	1.140416000000	−0.103343000000	1.097260000000
B	1.806969000000	1.963089000000	−0.386217000000
B	−1.542626000000	−1.932366000000	1.133568000000
Be	0.000000000000	1.577206000000	0.008758000000
B	−0.638597000000	−1.316233000000	2.228919000000

B	1.542626000000	1.932366000000	1.133568000000
B	0.000000000000	0.000000000000	2.724881000000
B	0.638597000000	1.316233000000	2.228919000000
Be	0.000000000000	−1.577206000000	0.008758000000
Be	−1.148876000000	0.082815000000	−1.070831000000
B	1.176139000000	1.697602000000	−1.775334000000
Be	1.148876000000	−0.082815000000	−1.070831000000
B	−1.806969000000	−1.963089000000	−0.386217000000
B	0.326002000000	0.702995000000	−2.591527000000
B	−1.176139000000	−1.697602000000	−1.775334000000
B	−0.326002000000	−0.702995000000	−2.591527000000

17

5.631185000 Be6B11_0008.out

B	1.528679000000	1.701700000000	0.509569000000
B	−0.117021000000	−0.964914000000	−1.615283000000
B	−1.648819000000	−0.707237000000	−1.161047000000
Be	−0.302031000000	−1.449462000000	0.175321000000
B	2.641512000000	−0.596366000000	−0.644848000000
Be	−1.789419000000	1.565781000000	0.309923000000
Be	1.619805000000	−0.697074000000	0.999108000000
B	−1.341198000000	−0.925353000000	1.696499000000
B	1.342610000000	−1.332495000000	−1.175056000000
Be	−0.127111000000	0.623224000000	1.414746000000
Be	−0.940410000000	0.987997000000	−1.391875000000
Be	1.010995000000	0.511028000000	−0.864484000000
B	−2.628326000000	0.474501000000	−0.801661000000
B	0.139389000000	−1.127775000000	1.950483000000
B	2.710593000000	0.706957000000	0.147077000000
B	0.040832000000	1.912171000000	0.081513000000
B	−2.245715000000	−0.374385000000	0.498565000000

17

10.209425000 Be6B11_0009.out

Be	1.784070000000	−0.881154000000	0.000000000000
Be	−1.178349000000	1.635847000000	0.000000000000
Be	1.064755000000	1.033056000000	0.000000000000
Be	−0.937588000000	−0.323485000000	1.174575000000
B	−2.310757000000	0.242038000000	0.000000000000
B	0.892147000000	−0.081631000000	−1.612675000000
B	0.352235000000	1.385244000000	1.718054000000
Be	−0.937588000000	−0.323485000000	−1.174575000000
Be	−0.817189000000	−2.391480000000	0.000000000000
B	0.892147000000	−0.081631000000	1.612675000000

B 0.621579000000 −1.643571000000 1.185587000000
B −2.180768000000 −1.259499000000 0.000000000000
B 0.352235000000 1.385244000000 −1.718054000000
B 0.285545000000 2.649292000000 0.765420000000
B 1.006026000000 −2.600648000000 0.000000000000
B 0.621579000000 −1.643571000000 −1.185587000000
B 0.285545000000 2.649292000000 −0.765420000000
17
11.904302500 Be6B11_0010.out
Be −0.967864000000 −0.003324000000 −2.025249000000
Be −0.977869000000 −0.908311000000 1.005310000000
Be 0.977869000000 0.908311000000 1.005310000000
Be 0.967864000000 0.003324000000 −2.025249000000
Be −1.631200000000 0.684878000000 −0.239010000000
B 0.932498000000 −0.979840000000 1.526020000000
B −0.932498000000 0.979840000000 1.526020000000
Be 1.631200000000 −0.684878000000 −0.239010000000
B 1.725675000000 1.166405000000 −0.793865000000
B 0.000000000000 −1.306175000000 −1.105711000000
B 0.000000000000 1.306175000000 −1.105711000000
B 0.440133000000 −2.107280000000 0.385744000000
B −1.725675000000 −1.166405000000 −0.793865000000
B −0.440133000000 2.107280000000 0.385744000000
B 0.995287000000 2.407450000000 −0.164110000000
B −0.995287000000 −2.407450000000 −0.164110000000
B 0.000000000000 0.000000000000 2.318162000000
17
14.065412500 Be6B11_0011.out
Be 2.136962000000 0.466122000000 −0.136821000000
Be −2.136962000000 −0.466122000000 −0.136821000000
Be 0.448793000000 1.453418000000 0.501781000000
Be −0.448793000000 −1.453418000000 0.501781000000
B −1.013219000000 −1.183852000000 −1.416467000000
B 1.013219000000 1.183852000000 −1.416467000000
B −1.516619000000 −0.303767000000 1.526878000000
Be 0.858236000000 −0.661623000000 −1.331966000000
Be −0.858236000000 0.661623000000 −1.331966000000
B 1.516619000000 0.303767000000 1.526878000000
B 0.000000000000 0.000000000000 1.865341000000
B 0.000000000000 2.333754000000 −1.105419000000
B 0.000000000000 −2.333754000000 −1.105419000000
B 1.546339000000 −1.193439000000 0.921284000000

B	−1.546339000000	1.193439000000	0.921284000000
B	−1.178288000000	2.314234000000	−0.085342000000
B	1.178288000000	−2.314234000000	−0.085342000000

17

25.153337500 Be6B11_0012.out

Be	−1.277429000000	1.666744000000	0.000000000000
B	−1.541731000000	−2.135511000000	0.000000000000
Be	−0.626280000000	−1.277275000000	1.405826000000
B	−0.006859000000	−2.441749000000	0.000000000000
Be	−0.626280000000	−1.277275000000	−1.405826000000
B	1.212038000000	−1.565687000000	0.808025000000
B	0.037720000000	1.982302000000	1.348142000000
B	−0.811599000000	0.576246000000	1.455720000000
B	1.777627000000	−0.269016000000	0.000000000000
Be	0.926136000000	0.082001000000	−1.658674000000
Be	0.926136000000	0.082001000000	1.658674000000
B	0.037720000000	1.982302000000	−1.348142000000
B	1.212038000000	−1.565687000000	−0.808025000000
Be	−1.878824000000	−0.366759000000	0.000000000000
B	−0.811599000000	0.576246000000	−1.455720000000
B	0.324901000000	2.740038000000	0.000000000000
B	0.614978000000	0.992966000000	0.000000000000

17

42.350602500 Be6B11_0013.out

Be	−0.000618000000	1.265154000000	−1.244622000000
Be	0.000618000000	−1.265154000000	−1.244622000000
B	−0.768950000000	−0.000057000000	−2.360234000000
Be	1.577107000000	0.000458000000	1.121963000000
B	0.001596000000	1.217530000000	1.359563000000
B	−1.321661000000	1.600756000000	0.207885000000
B	1.321661000000	−1.600756000000	0.207885000000
Be	−1.852297000000	−0.000057000000	−0.867758000000
B	1.322470000000	1.601879000000	0.207441000000
B	−1.322470000000	−1.601879000000	0.207441000000
B	0.768950000000	0.000057000000	−2.360234000000
B	0.000000000000	0.000000000000	2.355612000000
Be	−1.577107000000	−0.000458000000	1.121963000000
Be	1.852297000000	0.000057000000	−0.867758000000
B	0.000000000000	2.439167000000	0.199873000000
B	−0.001596000000	−1.217530000000	1.359563000000
B	0.000000000000	−2.439167000000	0.199873000000
