# Supplementary figures and images for: Exploration of Free Energy Surface and Thermal Effects on Relative Population and Infrared Spectrum of the Be6B11− Fluxional Cluster
Source: Materials (Basel). 2020 Dec 29;14(1):112. doi: 10.3390/ma14010112 (PMC7796227; doi:10.3390/ma14010112)

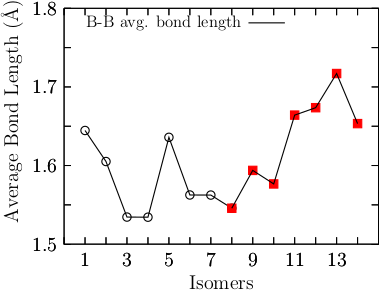

Supplement: Supplementary file 1 [file materials-14-00112-s001.zip › figures_and_Supplementary_Be6B11/figures_2.png]

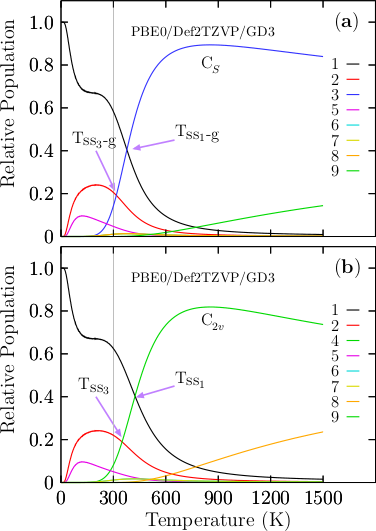

Supplement: Supplementary file 1 [file materials-14-00112-s001.zip › figures_and_Supplementary_Be6B11/figures_5.png]

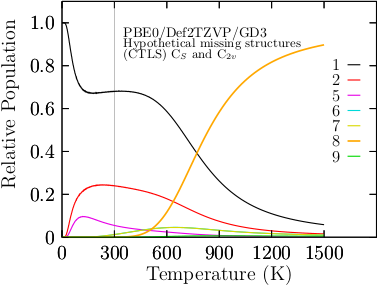

Supplement: Supplementary file 1 [file materials-14-00112-s001.zip › figures_and_Supplementary_Be6B11/figures_4.png]

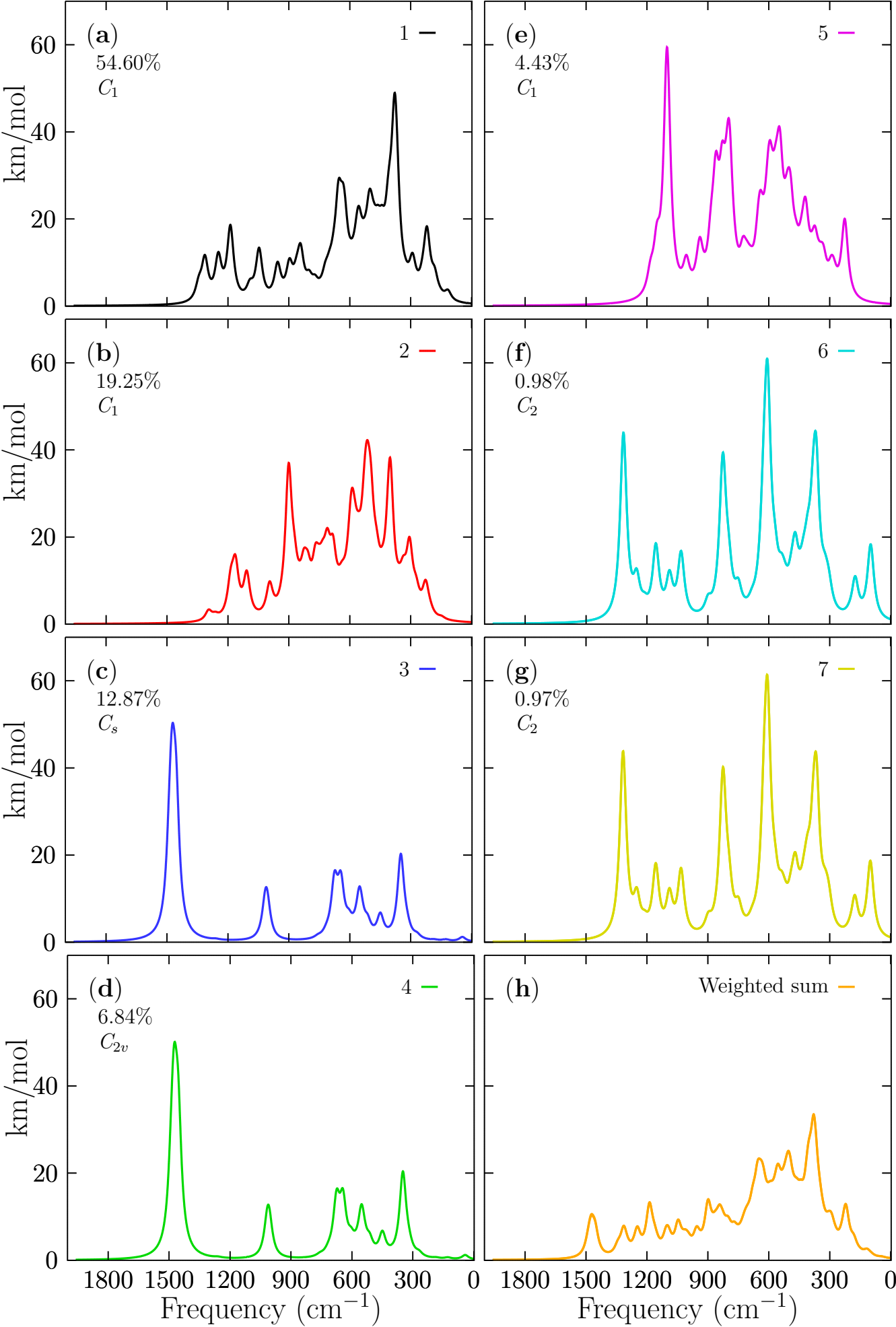

Supplement: Supplementary file 1 [file materials-14-00112-s001.zip › figures_and_Supplementary_Be6B11/figures_7.pdf]

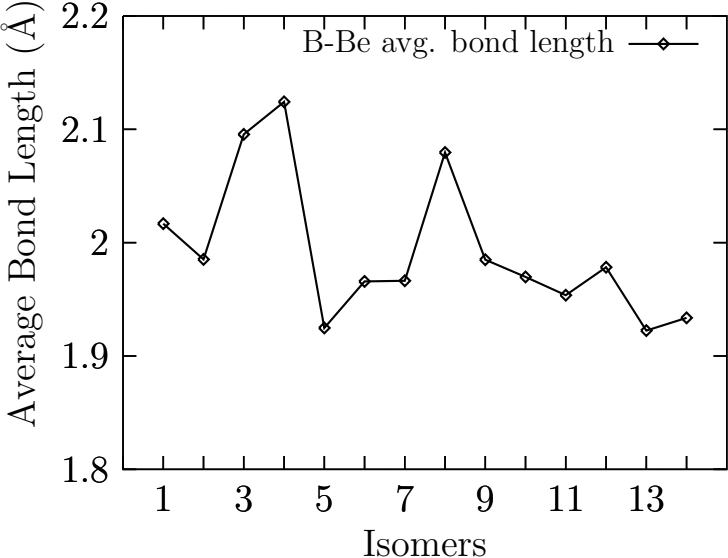

Supplement: Supplementary file 1 [file materials-14-00112-s001.zip › figures_and_Supplementary_Be6B11/figures_A1.pdf]

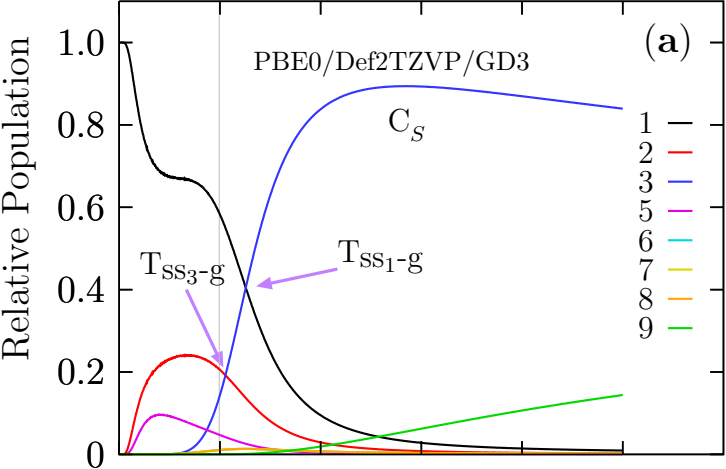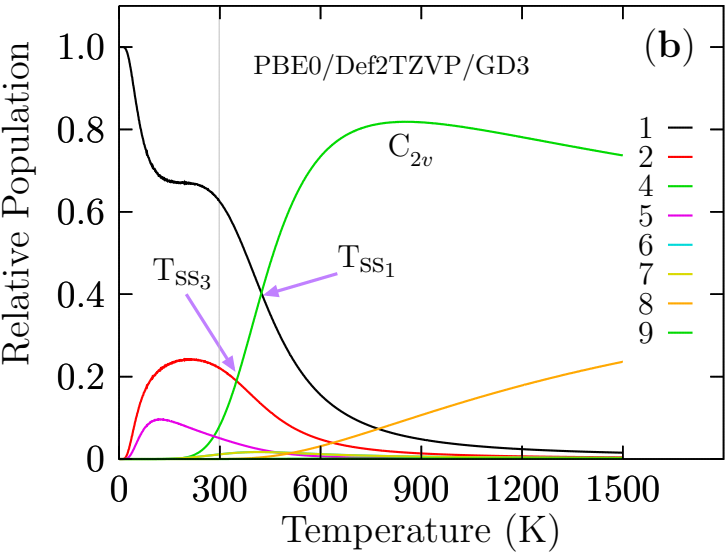

Supplement: Supplementary file 1 [file materials-14-00112-s001.zip › figures_and_Supplementary_Be6B11/figures_5.pdf]

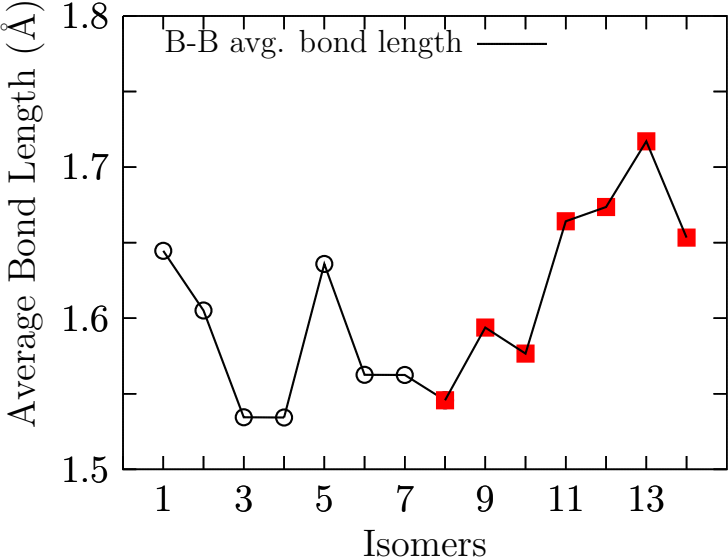

Supplement: Supplementary file 1 [file materials-14-00112-s001.zip › figures_and_Supplementary_Be6B11/figures_2.pdf]

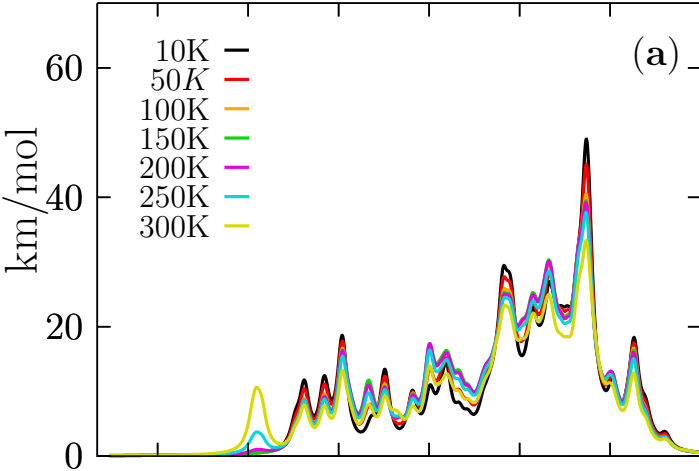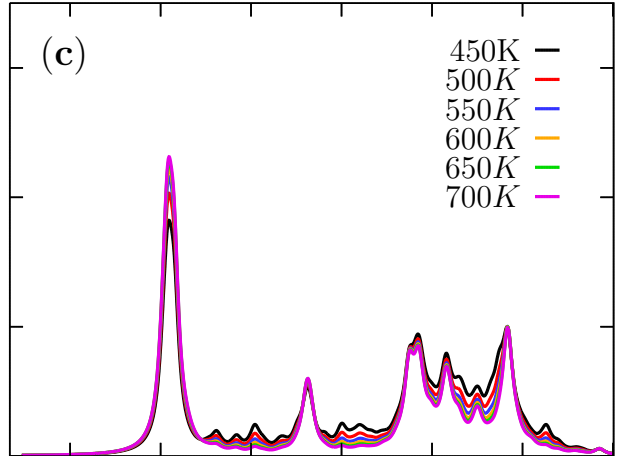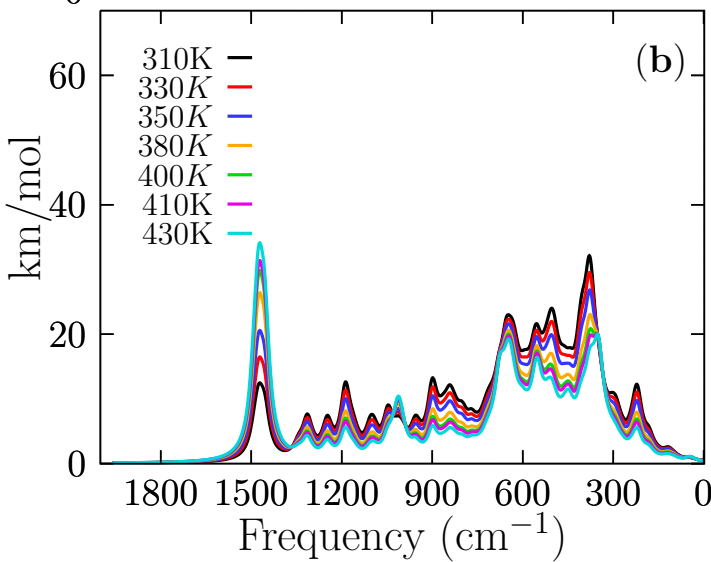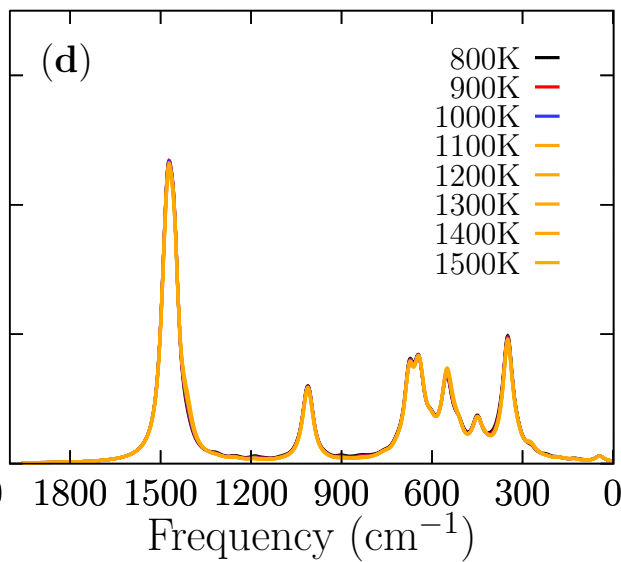

Supplement: Supplementary file 1 [file materials-14-00112-s001.zip › figures_and_Supplementary_Be6B11/figures_8.pdf]

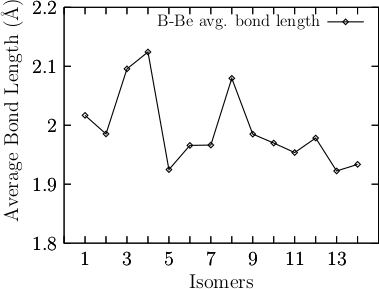

Supplement: Supplementary file 1 [file materials-14-00112-s001.zip › figures_and_Supplementary_Be6B11/figures_A1.png]

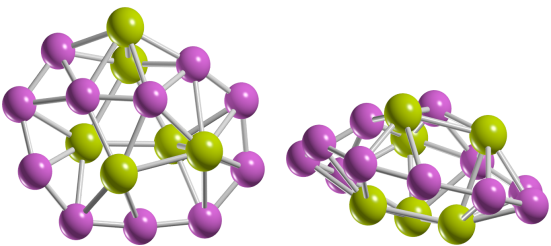

1(0.0)[**54.62%**]

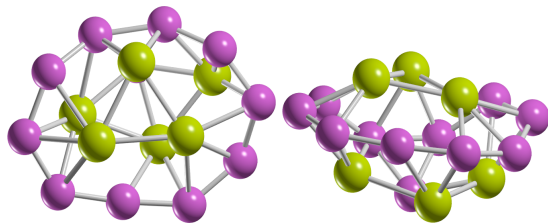

2(0.61)[**19.23%**]

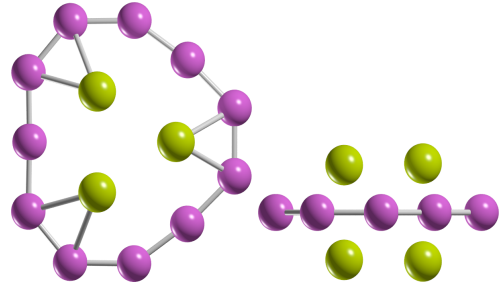

3(0.85)[**12.89%**]

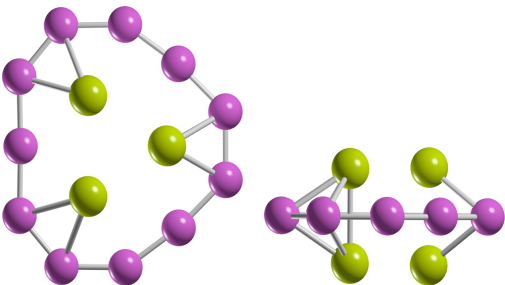

4(1.23)[**6.84%**]

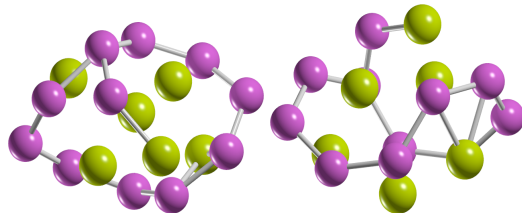

5(1.48)[**4.42%**]

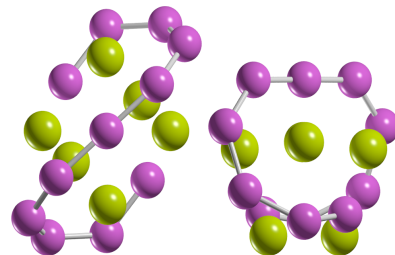

6(2.37)[**0.98%**]

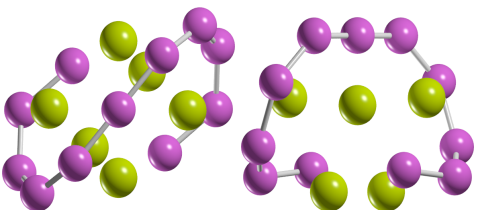

7(2.38)[**0.97%**]

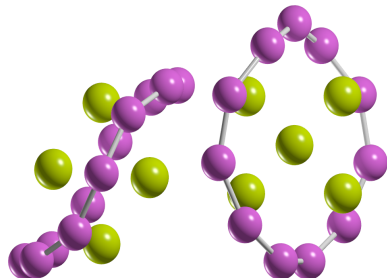

8(5.16)[**0.0%**]

Supplement: Supplementary file 1 [file materials-14-00112-s001.zip › figures_and_Supplementary_Be6B11/figures_1.pdf]

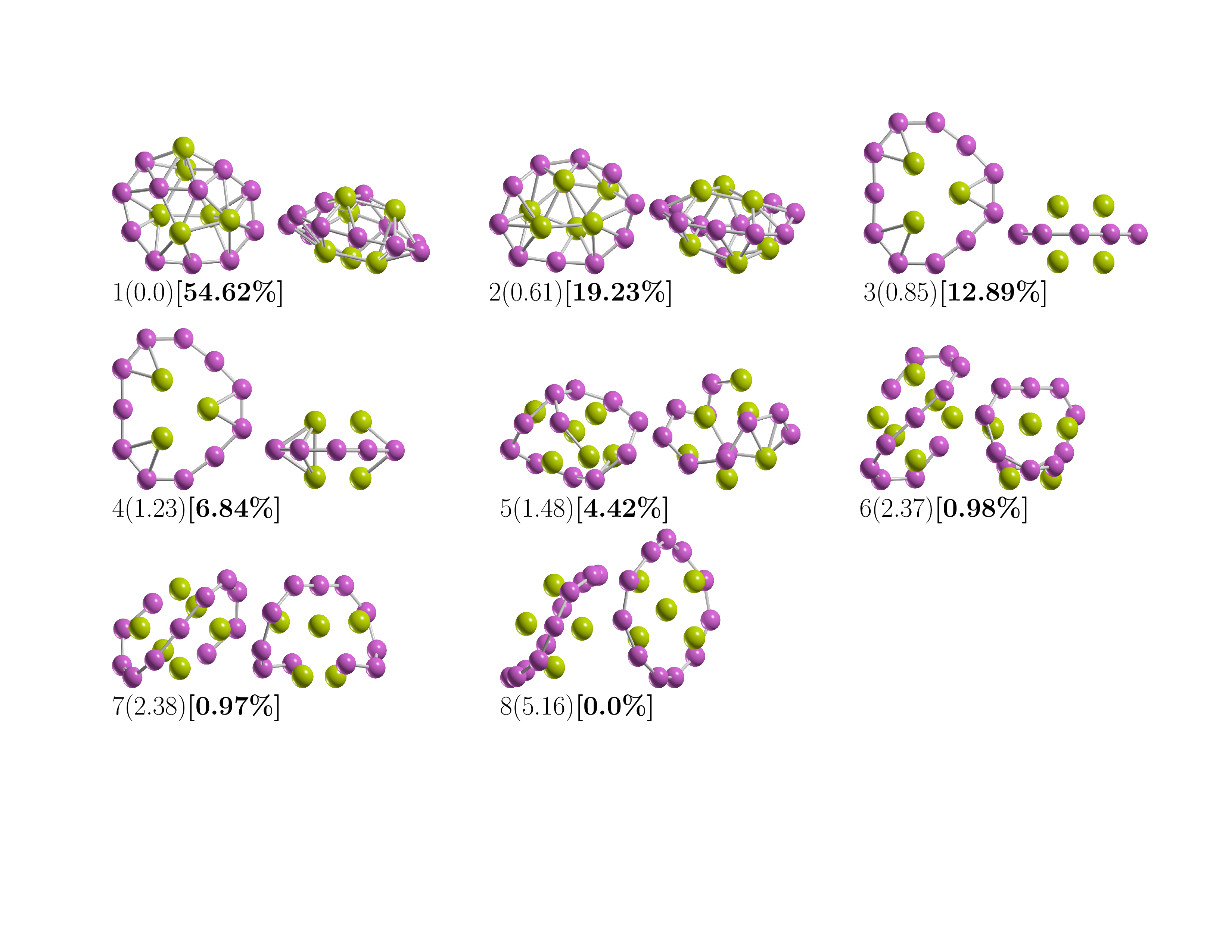

Supplement: Supplementary file 1 [file materials-14-00112-s001.zip › figures_and_Supplementary_Be6B11/figures_1.png]

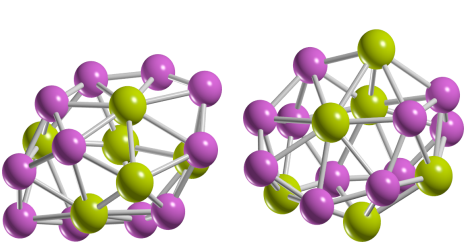

1(0.0)[**0.0**]

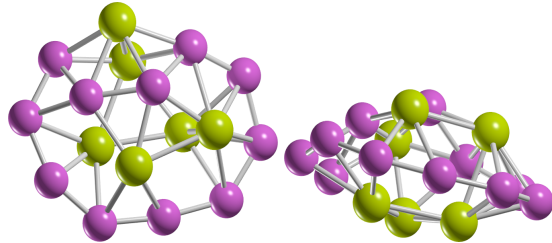

2(1.75)[**0.58**]

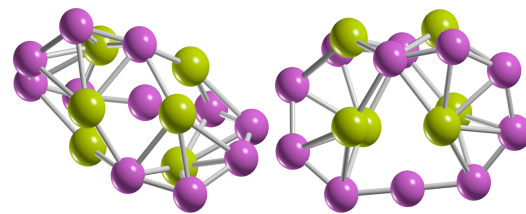

3(1.84)[**0.86**]

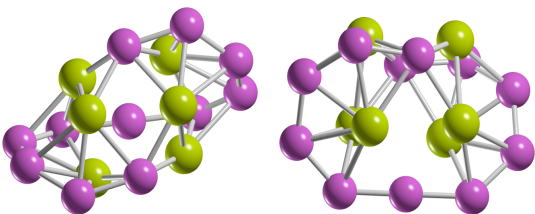

4(1.84)[**0.85**]

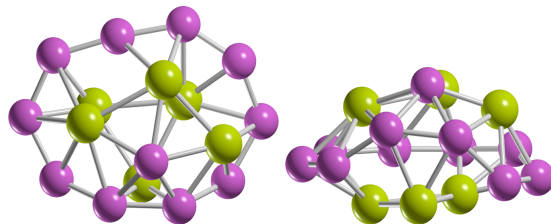

5(2.42)[**1.81**]

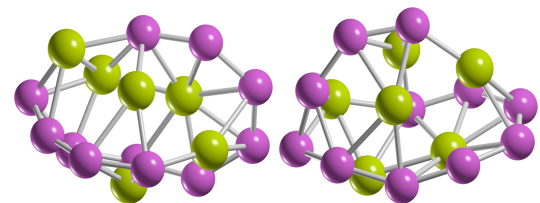

6(2.64)[**1.68**]

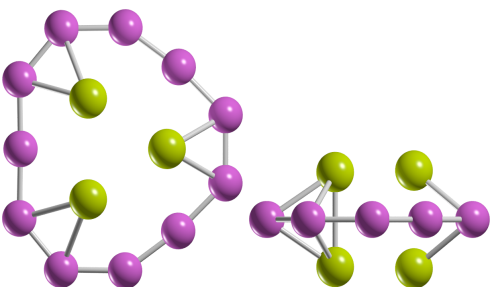

7(4.10)[**1.19**]

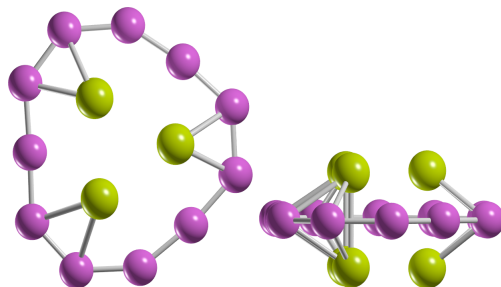

8(4.13)[**1.23**]

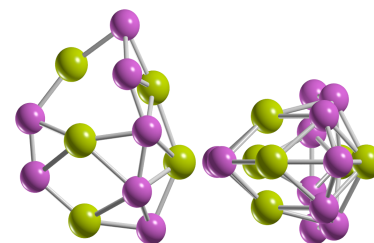

9(5.57)[**4.46**]

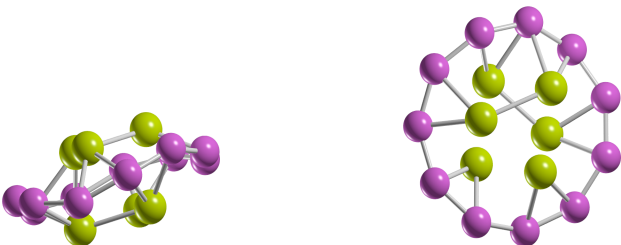

10(9.20)[**5.16**]

Supplement: Supplementary file 1 [file materials-14-00112-s001.zip › figures_and_Supplementary_Be6B11/figures_A2.pdf]

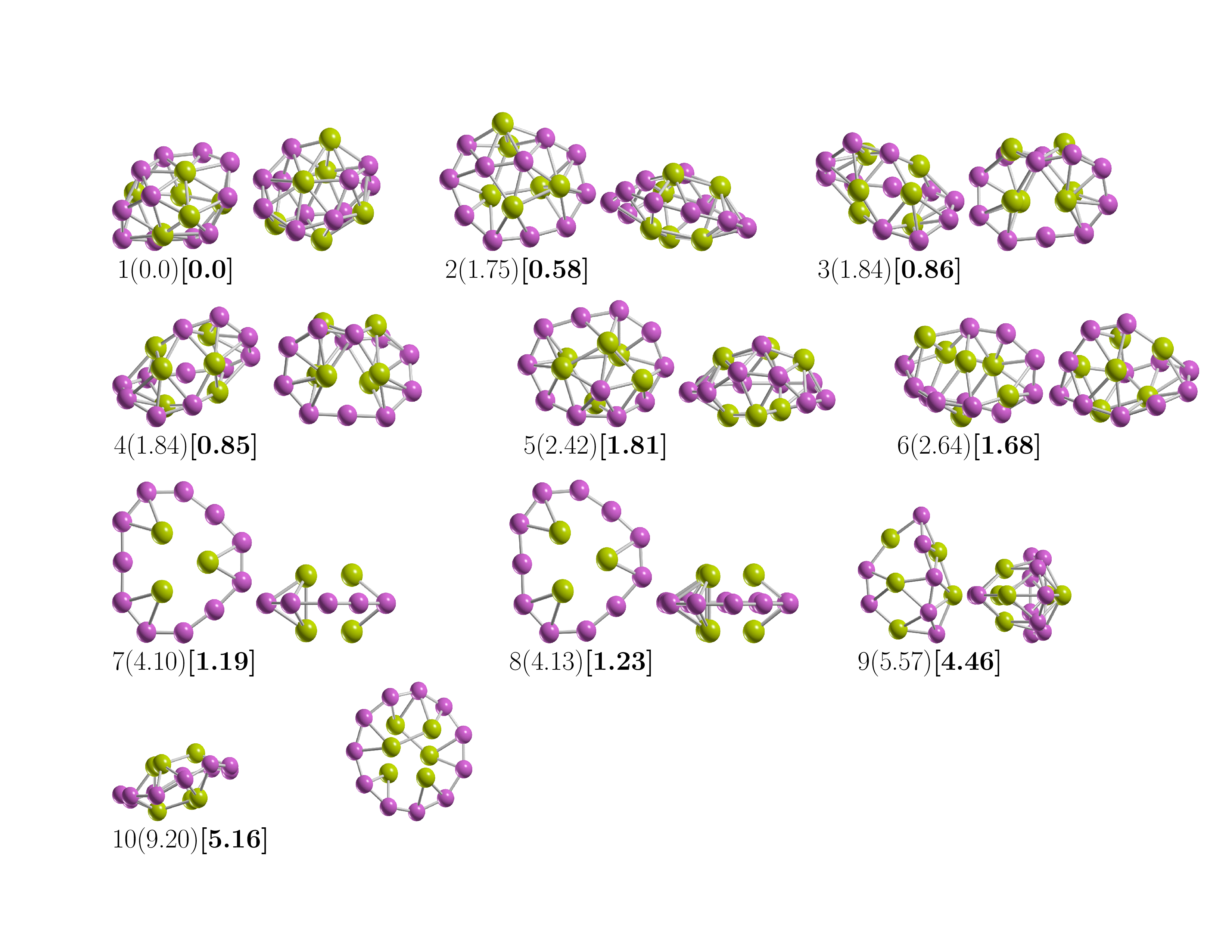

Supplement: Supplementary file 1 [file materials-14-00112-s001.zip › figures_and_Supplementary_Be6B11/figures_A2.png]

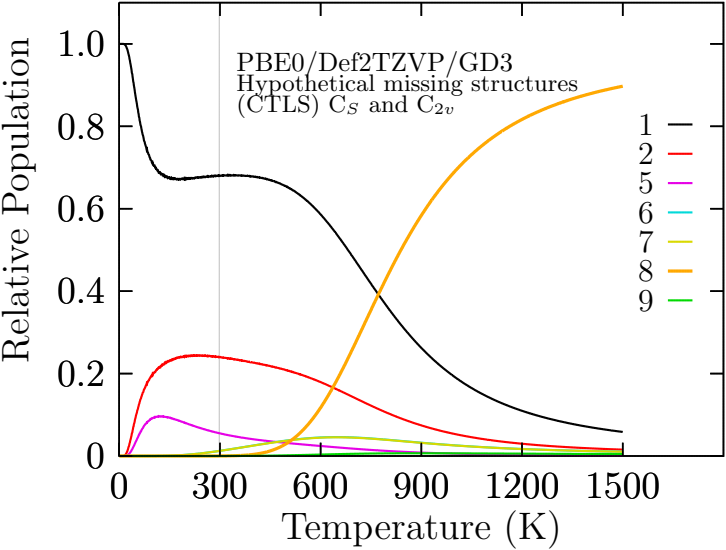

Supplement: Supplementary file 1 [file materials-14-00112-s001.zip › figures_and_Supplementary_Be6B11/figures_4.pdf]

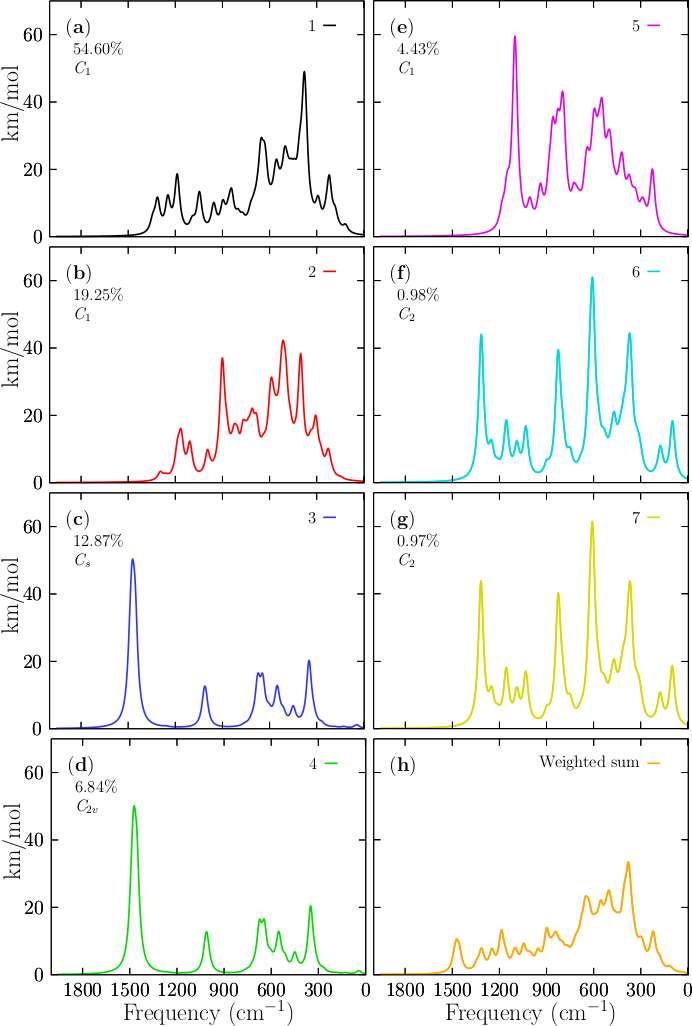

Supplement: Supplementary file 1 [file materials-14-00112-s001.zip › figures_and_Supplementary_Be6B11/figures_7.png]

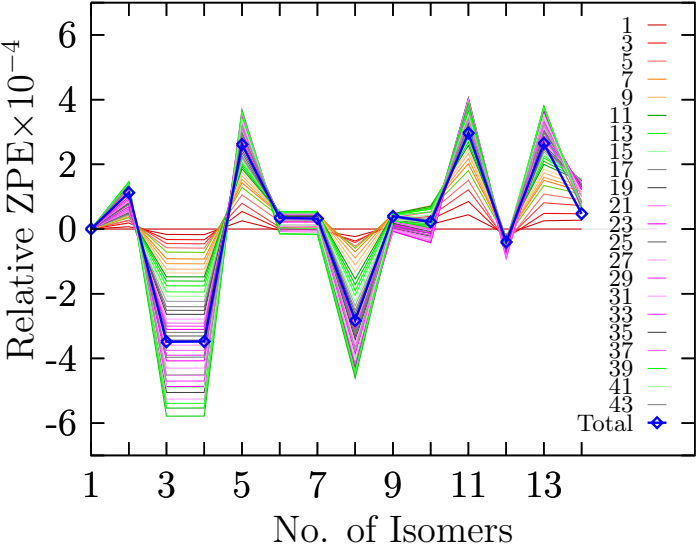

Supplement: Supplementary file 1 [file materials-14-00112-s001.zip › figures_and_Supplementary_Be6B11/figures_6.pdf]

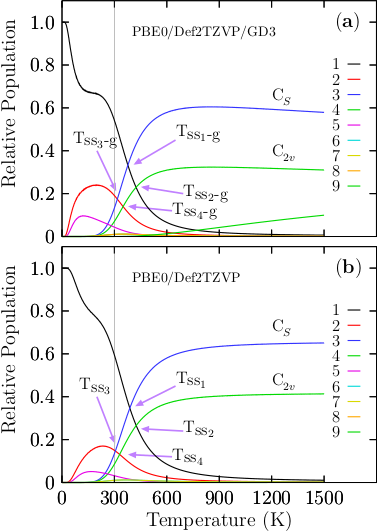

Supplement: Supplementary file 1 [file materials-14-00112-s001.zip › figures_and_Supplementary_Be6B11/figures_3.png]

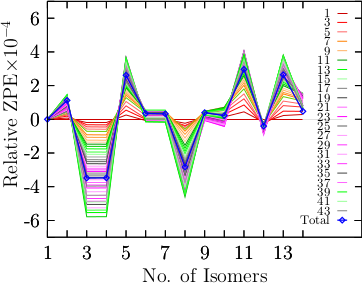

Supplement: Supplementary file 1 [file materials-14-00112-s001.zip › figures_and_Supplementary_Be6B11/figures_6.png]

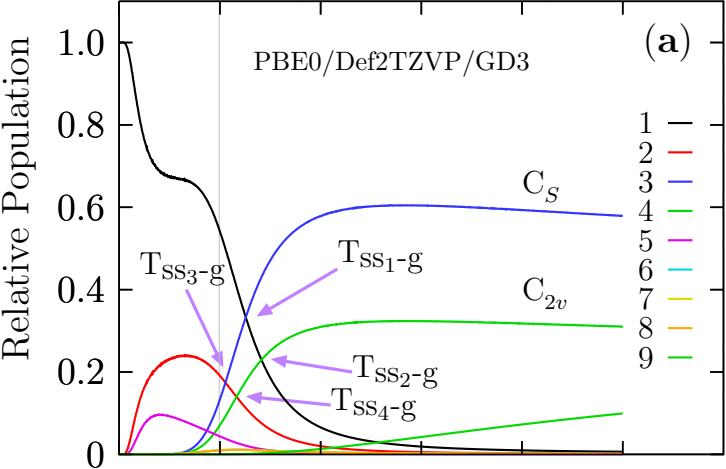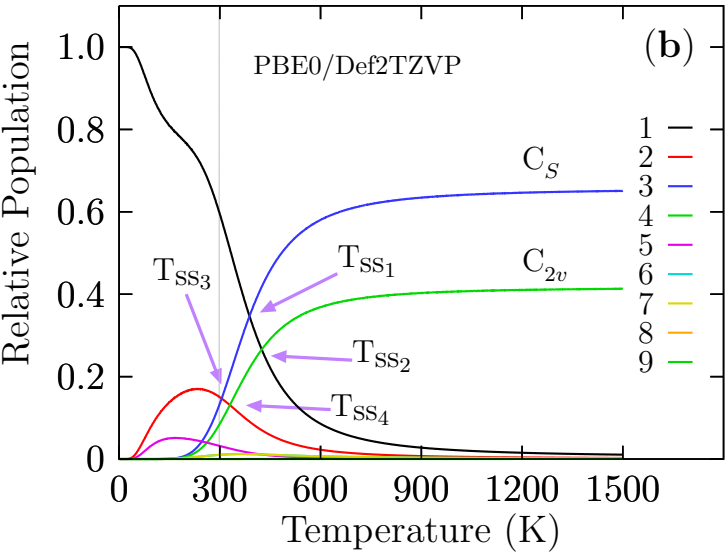

Supplement: Supplementary file 1 [file materials-14-00112-s001.zip › figures_and_Supplementary_Be6B11/figures_3.pdf]
